# Depletion of alloreactive B cells by drug-resistant chimeric alloantigen receptor T cells to prevent transplant rejection

**DOI:** 10.1016/j.ymthe.2025.01.009

**Published:** 2025-01-11

**Authors:** Anna Christina Dragon, Agnes Bonifacius, Stefan Lienenklaus, Murielle Verboom, Jan-Phillipp Gerhards, Fabio Ius, Christian Hinze, Michael Hudecek, Constanca Figueiredo, Rainer Blasczyk, Britta Eiz-Vesper

**Affiliations:** 1 Institute of Transfusion Medicine and Transplant Engineering, Hannover Medical School, 30625 Hannover, NI, Germany; 2 nextGENERATION Medical Scientist Program, Dean’s Office for Academic Career Development, Hannover Medical School, 30625 Hannover, NI, Germany; 3 Institute of Laboratory Animal Science, Hannover Medical School, 30625 Hannover, NI, Germany; 4 Department of Cardiothoracic-, Transplantation- and Vascular Surgery, Hannover Medical School, 30625 Hannover, NI, Germany; 5 Department of Nephrology and Hypertension, Hannover Medical School, 30625 Hannover, NI, Germany; 6 Cellular Immunotherapy, Department of Internal Medicine II, University Hospital of Wuerzburg, 97080 Wuerzburg, BY, Germany

**Keywords:** engineered T cells, antibody-mediated rejection, solid organ transplantation, transplant rejection, alloreactivity, chimeric alloantigen receptor, CAR-T cells, alloreactive B cells, HLA, mismatch

## Abstract

Antibody-mediated rejection (AMR) remains a major complication after solid organ transplantation (SOT). Current treatment options are inefficient and result in drastic impairment of the general immunity. To selectively eliminate responsible alloreactive B cells characterized by anti-donor-HLA B cell receptors (BCRs), we generated T cells overcoming rejection by antibodies (CORA-Ts) engineered with a novel chimeric receptor comprising a truncated donor-HLA molecule as antigen recognition domain. As proof-of-concept, CORA receptors based on HLA-A∗02 were developed. In co-cultures with anti-HLA-A∗02 B cell lines, CORA-Ts were specifically activated, released pro-inflammatory mediators, and exhibited strong cytotoxicity resulting in an effective reduction of anti-HLA-A∗02 antibody release. Significant reduction of growth of an anti-HLA-A∗02 B cell line could be confirmed using an *in vivo* mouse model. Modification of the CORA receptor effectively abrogated T cell binding, thereby avoiding T cell sensitization. Additionally, using CRISPR-Cas9-mediated knockout of the *FKBP12* gene, CORA-Ts were able to resist immunosuppressive treatment with tacrolimus, thereby allowing high efficiency in transplant patients. Our results demonstrate that CORA-Ts are able to specifically eliminate alloreactive, anti-HLA B cells, thus selectively preventing anti-HLA antibody release even under immunosuppressive conditions. This suggests CORA-Ts as potent approach to combat AMR and improve long-term graft survival in SOT patients while preserving their overall B cell immunity.

## Graphical abstract

**Figure:**
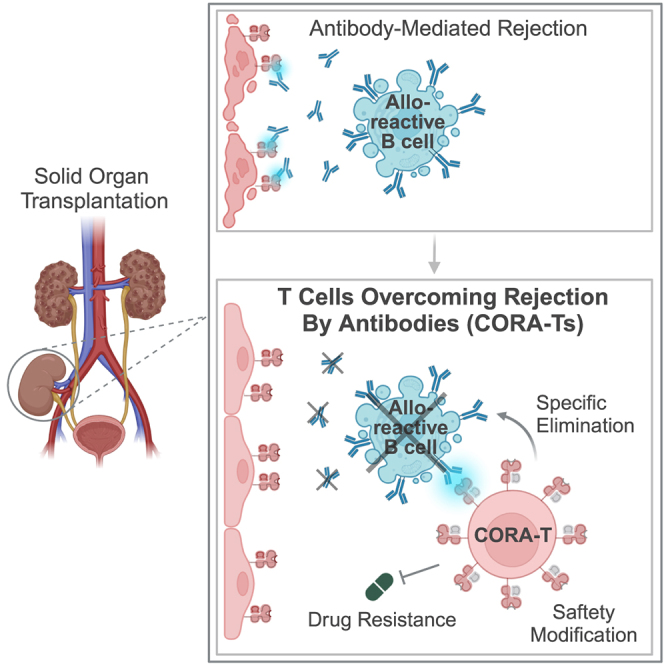

## Teaser

Eiz-Vesper and colleagues developed a promising novel cell therapeutic approach to selectively combat antibody-mediated rejection, a serious and unresolved complication after solid organ transplantation. T cells overcoming rejection by antibodies (CORA-Ts) were demonstrated to effectively eliminate responsible alloreactive B cells, restrict anti-HLA antibody release, and maintain their functionality under immunosuppression.

## Introduction

The only effective treatment of end-stage organ diseases is replacement of the respective organ by solid organ transplantation (SOT). However, one of the major challenges after SOT is antibody-mediated rejection (AMR), which is considered an outstanding question in transplantation^1^ and recognized to be even more important than T cell-mediated rejection (TMR) as the most common reason of late allograft failure.^2^ In most cases, donor-specific antibodies (DSAs) directed against mismatched HLA class I and II molecules can be detected in the blood and bind to graft endothelial cells.^3^ Clinically, this is evident by detection of complement deposition, microvascular inflammation with leukocyte infiltration, and endothelial injury, finally leading to irreversible tissue injury and premature allograft failure.^4^ A recent meta-analysis evaluating various follow-up studies of kidney transplantation assessed an incidence of 1%–22% for acute AMR and 8%–20% for chronic AMR up to 10 years after transplantation.^2^ The incidence is even higher for patients transplanted with other organs or with preformed DSAs.

Modern immunosuppressive treatment mainly addresses TMR, which only indirectly affects the B cell alloimmune response, as the development of antibodies requires the help of T cells. Studies showed that such maintenance therapy has only little effects on plasma cells and memory B cells, thereby solely delaying the formation of DSAs.^4^ This led to the development of therapeutic approaches directly targeting B cells and interfering with DSA-mediated destruction cascades, such as depletion of all B cells by the anti-CD20 antibody rituximab. However, no clear clinical benefit of B cell depletion in chronic AMR could be proven in randomized controlled trials.^3^ This is hypothesized to be due to the concomitant elimination of regulatory B cell (B_reg_) subpopulations with beneficial effects for allograft tolerance.^5^ Further therapeutic approaches have been tested; however, randomized studies are lacking or not showing significant effects so far, so that there is, until now, no effective treatment in late and chronic AMR.^3^ Moreover, unspecific interference with general B cell functionality in all of these approaches is associated with an increased infection risk in the already immunocompromised transplant recipients, emphasizing the need for a more precise targeting of alloreactive B cells.

T cells engineered with a chimeric antigen receptor (CAR-Ts) widely emerged as a therapeutic approach to selectively target and eliminate malignant cells in context of different diseases. In hematological malignancies, CAR-Ts exhibit impressive antitumor activity prompting their clinical approval in different indications.^6^ Using a similar approach, we aimed to selectively target anti-donor-HLA B cells responsible for AMR via their respective BCRs in the current study to address both emerging alloreactive naive as well as pre-existing alloreactive memory B cells in the patient. As a proof-of-concept, we redirected T cells toward alloreactive anti-HLA-A∗02 B cells by introducing a novel CAR-like receptor in order to generate chimeric alloantigen-specific T cells overcoming rejection by antibodies (CORA-Ts). Upon recognition of B cells harboring anti-HLA-A∗02 BCRs, CORA-Ts were specifically activated and mediated elimination of these B cells, resulting in an efficient reduction of anti-HLA-A∗02 antibody release. Specific reduction of the growth of anti-HLA-A∗02 B cells could moreover be confirmed in a preclinical *in vivo* model and in *in vitro* triple co-cultures with BCR-expressing and antibody-releasing B cell mixtures of different specificities. As important steps toward the clinical application of CORA-Ts, we have integrated a modification preventing binding and sensitization of alloreactive T cells, as well as an additional modification allowing preservation of effector functionality under immunosuppressive conditions, thus generating tacrolimus-resistant CORA-Ts. Based on these promising results, application of CORA-Ts might serve as an innovative approach to specifically combat AMR caused by anti-donor-HLA B cells and effectively improve long-term graft survival in SOT.

## Results

### CORA_sh receptors comprise a properly assembled HLA-A∗02 complex

**Figure 1:**
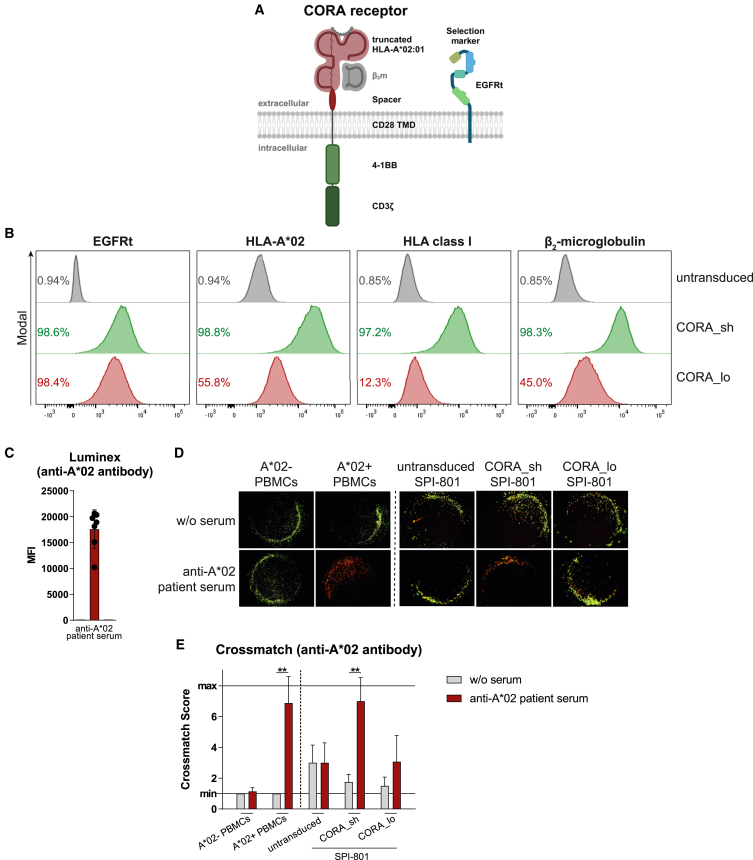
CORA receptors comprising a truncated HLA-A∗02 complex mediate recognition of patient-derived anti-HLA-A∗02 DSAs (A) CORA receptors comprise HLA-A∗02:01 chains α_1_-α_3,_ either a short (sh) or a long (lo) spacer domain, a transmembrane domain (TMD) of CD28 and the intracellular signaling domains of 4-1BB and CD3ζ. β_2_-microglobulin (β_2_m) is not encoded in the vector. A co-expressed truncated epidermal growth factor receptor (EGFRt) serves as transduction and selection marker. (B) CORA_sh and CORA_lo receptors were expressed in SPI-801 cells and detected by flow cytometry as shown in representative histograms. Untransduced SPI-801 cells served as control. (C) Presence of anti-HLA-A∗02 antibody in the serum of kidney transplant recipients was detected by Luminex. Data are shown as scattered dot plot with mean ± SD, whereby each symbol represents an individual patient (*n* = 7). (D and E) Sera of the same patients were used in crossmatch assays with HLA-A∗02-negative or -positive PBMCs from healthy donors, as well as SPI-801 cells transduced with CORA receptors. (D) Representative pictures and (E) crossmatch scores indicate CDC based on evaluation of viable cells (green) vs. dead cells (red) after complement addition. (E) Data are shown as mean + SD (*n* = 4–7). Statistical analysis was performed by using Mann-Whitney test. ∗∗*p* ≤ 0.01, ∗∗∗*p* ≤ 0.001.

As alloreactive B cells releasing antibodies with anti-donor-HLA specificity are responsible for AMR and thus the most common reason for late allograft failure,^2^ we developed a cell therapeutic approach to selectively deplete responsible anti-HLA B cells. As proof-of-concept, CORA receptors consisting of a truncated HLA-A∗02 α-chain connected to intracellular 4-1BB and CD3ζ-signaling domains were designed to specifically target anti-HLA-A∗02 B cells via their anti-HLA-A∗02 BCR (Figure 1A). CORA receptor variants differing in their spacer length (CORA_sh with a 12 amino acid [aa] or CORA_lo with a 229 aa spacer domain) were generated to determine the construct with superior HLA assembly and functional abilities. To evaluate correct expression and folding of the HLA-A∗02 molecule within the chimeric receptor, both CORA receptors were transduced into HLA-negative SPI-801 cells, followed by enrichment of transduced cells via a co-expressed truncated epidermal growth factor receptor (EGFRt) serving as selection marker (Figures 1B and S1). By flow cytometry, all CORA_sh^+^ cells could be stained for HLA-A∗02 and cell-endogenous β_2_-microglobulin (β_2_m) compared with untransduced SPI-801 cells that did not express either molecule (Figures 1B and S1). On CORA_lo^+^ SPI-801 cells, HLA-A∗02 and β_2_m were detectable on 56% and 45% of cells, respectively. As indicated by binding of the conformational anti-HLA class I antibody W6/32, CORA_sh receptors comprised a correctly folded HLA-A∗02-complex loaded with peptide on all cells, whereas CORA_lo receptors were only expressed with the complete HLA complex on 12% of cells, thus indicating that the long spacer impaired complete association of the HLA molecule with β_2_m and peptide by the cell-endogenous machinery (Figures 1B and S1).

### CORA receptors mediate recognition of patient-derived anti-HLA-A∗02 DSAs

To evaluate binding of patient-derived anti-HLA-A∗02 antibodies to CORA receptors, sera from seven kidney transplant recipients were selected, from which four had anti-HLA-A∗02 DSAs and three had anti-HLA-A∗02 antibodies independent from the transplant HLA type. In all seven patient sera, detectable concentrations of anti-HLA-A∗02 antibodies could be measured by using Luminex, a multiplexed assay with HLA allele-coupled beads widely used for clinical proof of anti-HLA DSAs^7^ (Figure 1C). In a crossmatch, these antibodies were examined for their ability to bind to the generated CORA receptors (Figures 1D and 1E). As expected, anti-HLA-A∗02 antibodies present in sera of these patients bound to peripheral blood mononuclear cells (PBMCs) from HLA-A∗02-positive healthy individuals and induced complement-dependent cytotoxicity (CDC) as indicated by a significantly increased crossmatch score^8^ of 7 (dead cells 40%–100%). The same sera did not induce CDC toward HLA-A∗02-negative PBMCs (score = 1; dead cells≤10%) indicating anti-HLA-A∗02 specificity. Following addition of anti-HLA-A∗02 patient sera to SPI-801 cells transduced with CORA_sh receptors, significantly increased CDC (score = 6; dead cells 40%–80%) was induced, whereas CDC was not increased following addition to untransduced SPI-801 cells. For CORA_lo^+^ SPI-801 cells, anti-HLA-A∗02 patient serum slightly increased CDC (score = 3; dead cells 10%–40%).

Taken together, transduction of CORA receptors comprising a truncated HLA-A∗02 α-chain enabled correct assembly of the HLA-peptide complex by the cell-endogenous machinery when connected with a short spacer domain. Such CORA_sh receptors bound to anti-HLA-A∗02 DSAs in sera of transplant patients suggesting their suitability to also target the respective membrane-bound BCRs with the same specificity.

### Hybridoma cells serve as surrogates for anti-HLA B cells releasing anti-HLA DSAs

**Figure 2:**
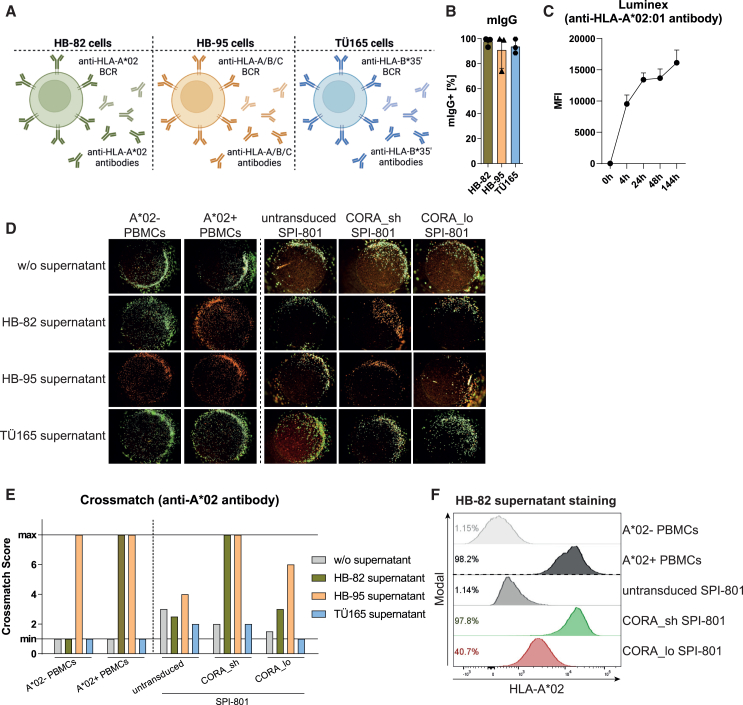
Hybridoma cells serve as surrogates for anti-HLA B cells releasing anti-HLA DSAs (A) HB-82 (anti-HLA-A∗02), HB-95 (anti-HLA-A/B/C), and TÜ165 (anti-HLA-B∗35ʹ loaded with LPPHDITPY) cells were used as a model for anti-HLA-antibody-releasing B cells. Created with BioRender.com. (B) Surface expression of BCRs was determined by flow cytometry and usage of anti-mouse immunoglobulin G (mIgG) antibody. Data are shown as scattered dot plot with mean ± SD, whereby each symbol represents an independent experiment (*n* = 3). (C) Release of anti-HLA-A∗02 antibody by HB-82 cells was detected by Luminex (*n* = 3–10). (D and E) Binding of anti-HLA antibodies present in the supernatant of hybridoma cells to HLA-A∗02-negative or -positive PBMCs from healthy donors (all HLA-B∗35-negative), as well as to SPI-801 cells transduced with CORA receptors was assessed by their ability to mediate CDC in crossmatch assays. (D) Representative pictures and (E) crossmatch scores indicate CDC based on evaluation of viable cells (green) vs. dead cells (red) after complement addition. Respective cells incubated without (w/o) supernatant served as viable controls (*n* = 1). (F) Cell culture supernatant of HB-82 cells containing anti-HLA-A∗02 antibody was used to stain HLA-A∗02-negative or -positive PBMCs from healthy donors, as well as CORA_sh^+^ or CORA_lo^+^ SPI-801 cells. MFI: mean fluorescence intensity.

As targets for evaluation of the functionality and specificity of CORA-Ts, hybridoma cells releasing anti-HLA-A∗02 (HB-82), anti-HLA-A/B/C (HB-95), or anti-HLA-B∗35 antibodies (TÜ165) were used and could be shown to express membrane-bound BCRs on 91%–98% of cells with comparable mean fluorescent intensities (MFIs) (Figures 2A, 2B, and S2A). Confirming their specificity, HB-82 and HB-95 but not TÜ165 cells could be stained with eukaryotic HLA-A∗02 multimer loaded with a cytomegalovirus (CMV)-derived peptide (Figure S2B). By Luminex, anti-HLA-A∗02 antibodies released by HB-82 cells were detectable in the cell culture supernatant after a short cultivation period and accumulated over time (Figure 2C). Binding specificity of secreted antibodies of all hybridoma cells was confirmed via crossmatch (Figures 2D and 2E). Similar to anti-HLA-A∗02 patient sera, the supernatant from HB-82 cells induced CDC toward CORA_sh^+^ SPI-801 cells in the same extent as toward HLA-A∗02-positive PBMCs (crossmatch scores = 8; dead cells 80%–100%), and crossmatch scores were also slightly increased for CORA_lo^+^ SPI-801 cells (scores = 3–4). In line, staining with HB-82 supernatant in flow cytometry revealed antibody binding to 98% of CORA_sh^+^ SPI-801 cells, and to 41% of CORA_lo^+^ SPI-801 cells (Figures 2F, S2C, and S2D). Antibodies present in the supernatant of HB-95 cells revealed the same specificity toward CORA receptors, whereas, as expected, antibodies released by TÜ165 cells did not induce more CDC when compared with respective cells cultured without supernatant (Figures 2D and 2E). Thus, the selected hybridoma cells are suitable surrogates for respective anti-HLA B cells releasing specific anti-HLA DSAs.

### CORA_sh-Ts mediate effective and target-specific T cell signaling, activation, cytokine expression, and cytotoxicity

**Figure 3:**
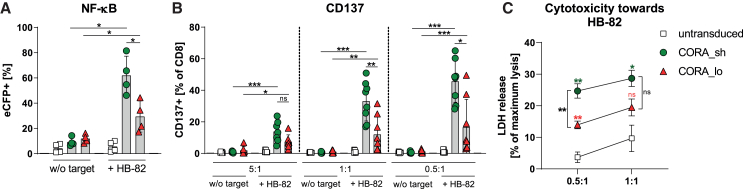
CORA_sh-Ts mediate effective and target-specific T cell signaling, activation and cytotoxicity CORA receptors with either a short (CORA_sh) or long (CORA_lo) spacer domain were transduced into (A) Jurkat-based reporter cells or (B and C) primary CD8^+^ T cells. Respective untransduced cells served as controls. (A) After cultivation of transduced reporter cells without (w/o) target cells or with HB-82 cells (anti-HLA-A∗02) in an E:T ratio of 1:1 for 24 h, transcription factor activity was determined by evaluation of NF-κB-induced enhanced cyan fluorescent protein (eCFP) reporter expression by flow cytometry (*n* = 4). (B) After co-cultivation of transduced CD8^+^ T cells with HB-82 cells in the indicated E:T ratios for 48 h, expression of CD137 as activation marker was assessed by flow cytometry (*n* = 6–8). (C) Cytotoxicity by CORA-Ts was assessed by LDH assay. Data are shown as mean ± SD (*n* = 5). Green and red asterisks indicate comparisons of CORA_sh- and CORA_lo-Ts, respectively, with untransduced T cells. (A–C) Data are shown as scattered dot plot with mean ± SD, whereby each symbol represents an independent donor. Statistical analysis was performed by using Mann-Whitney test. ns: not significant, ∗*p* ≤ 0.05, ∗∗*p* ≤ 0.01, ∗∗∗*p* ≤ 0.001.

To assess the ability of the CORA receptors to recognize anti-HLA-A∗02 B cells and initiate T cell signaling, they were transduced into an established Jurkat-derived reporter cell line indicating nuclear factor “k-light-chain-enhancer” of activated B cells (NF-κB) and nuclear factor of activated T cells (NFAT) activation, by upregulation of reporter fluorescent proteins detectable by flow cytometry.^9^ Following co-culture of either CORA_sh^+^ or CORA_lo^+^ reporter cells with the anti-HLA-A∗02 HB-82 cells, activity of both transcription factors was significantly upregulated, whereby the CORA_sh receptor mediated a more pronounced activation (Figures 3A and S3A). Both receptors were then transduced into primary CD8^+^ T cells isolated from healthy individuals, whereby presence of correctly assembled HLA-A∗02 after enrichment via co-expressed EGFRt was confirmed on 77% of CORA_sh-Ts and 36% of CORA_lo-Ts, respectively (Figures S3B and S3C). Following co-culture with HB-82 cells, the expression of CD25, CD69, and CD137 as indicators of T cell activation was significantly increased on CORA_sh- and CORA_lo-Ts in a ratio-dependent manner when compared with respective T cells cultured without target cells (Figures 3B, S3D, and S3E). In that, the CORA_sh receptor mediated significantly higher T cell activation than the CORA_lo receptor. In addition, intracellular expression of the effector molecules tumor necrosis factor (TNF-)α and granzyme B in CORA-Ts was increased in the same target-specific and ratio-dependent manner (Figures S3F and S3G). Importantly, both CORA_sh- and CORA_lo-Ts mediated significant cytotoxicity toward HB-82 cells when compared with untransduced T cells as indicated by an elevated release of lactate dehydrogenase (LDH) into the supernatant, whereby CORA_sh-Ts eliminated HB-82 cells significantly more efficiently when compared with CORA_lo-Ts (Figure 3C).

Thus, CORA receptors with a truncated HLA-A∗02 molecule as recognition domain mediate significant effector functionality of engineered T cells toward target cells expressing anti-HLA-A∗02 BCRs. In all performed functionality assays, CORA_sh-Ts showed significantly improved effector functions when compared with CORA_lo-Ts, which is in line with their improved expression of correctly assembled HLA-A∗02 complex as part of the CORA receptor. Hence, the subsequent experiments were focused on CORA_sh-Ts.

### Modification of the CORA_sh receptor prevents activation and proliferation of CD8^+^ T cells

Application of CORA-Ts based on HLA-A∗02 as recognition domain to eliminate anti-HLA-A∗02 DSA-producing B cells in HLA-A∗02-negative patients could potentially induce an additional sensitization of the patient’s T cells toward the HLA-A∗02 within the CORA receptor. To prevent that, we generated a CORA_sh receptor variant modified to abrogate binding of CD8^+^ T cells (CORA_sh_mod). For that, we exchanged two amino acids in the HLA-A∗02 α_3_-domain (D227K and T228A) described to markedly reduce activation of CD8^+^ T cells by abrogation of CD8 binding to HLA-A∗02.^10^ To confirm correct assembly of the modified HLA-A∗02 α-chain with cell-endogenous β_2_m and peptide, the CORA_sh_mod receptor was transduced into SPI-801 cells. All evaluated parameters, including detection of the correctly assembled HLA-A∗02 complex on CORA_sh_mod^+^ SPI-801 cells, as well as recognition of the receptor by anti-HLA-A∗02 DSAs from patient samples or by anti-HLA-A∗02 antibodies from hybridoma cell supernatants (Figure S4), revealed similar results when compared with CORA_sh^+^ SPI-801 cells (Figures 1, 2, S1, and S2). Moreover, CORA_sh_mod^+^ and CORA_sh^+^ SPI-801 were not recognized by patient samples containing various anti-HLA antibodies except HLA-A∗02 antibodies underlying their specificity. Thus, modification of the HLA-A∗02 domain to abrogate T cell binding did not influence expression and folding of the HLA molecule.

**Figure 4:**
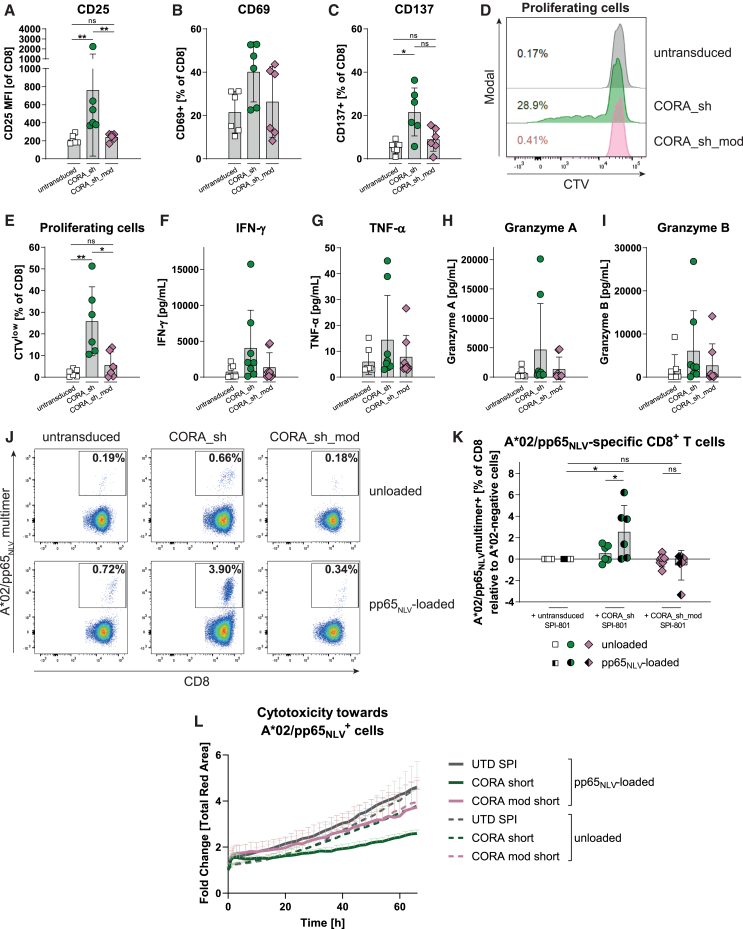
Modification of the CORA_sh receptor prevents activation and proliferation of CD8^+^ T cells CORA_sh receptors comprising either a truncated wild-type or a modified (D227K, T228A; CORA_sh_mod) HLA-A∗02 molecule were transduced into SPI-801 cells, whereby respective HLA-A∗02 domains are expected to dimerize with β_2_m and to be loaded with SPI-801-derived peptides. (A–K) CD8^+^ T cells were isolated from HLA-A∗02^+^ donors and co-cultured with untransduced or transduced SPI-801 cells in an E:T ratio of 1:1 for 7 days. (A–C) Expression of activation markers and (D and E) proliferation of CD8+ T cells as (D) representative histograms or (E) mean ± SD was assessed by flow cytometry. (F–I) Release of soluble mediators into the supernatant was assessed by LEGENDplex. (J–L) Before co-culture with CD8^+^ T cells, transduced SPI-801 cells were exogenously loaded with the HLA-A∗02-restricted, and CMVpp65-derived peptide NLVPMVATV. (F and G) After co-culture, the frequency of expanded HLA-A∗02/pp65_NLV_-specific T cells was assessed by multimer staining using flow cytometry and is shown as (F) representative dot plots or (G) relative values, whereby respective frequencies of HLA-A∗02/pp65_NLV_-specific T cells present in co-cultures with untransduced SPI-801 cells were subtracted from all values. (L) For evaluation of cytotoxicity toward transduced and pp65_NLV_-loaded SPI-801 cells, they were transduced to express mCherry before and co-cultured with HLA-A∗02/pp65_NLV_-specific T cells enriched from CD8^+^ of HLA-A∗02-positive donors using multimer. Live-cell imaging using the Incucyte Live-Cell Analysis System (Sartorius) was performed to evaluate elimination of mCherry-labeled cells. Total red object areas were analyzed by using the Incucyte software (Sartorius) and normalized to the time point of T cell addition, respectively. Data are shown as mean + SEM (*n* = 3). (A–C, E–I, K) Data are shown as scattered dot plot with mean ± SD, whereby each symbol represents an independent donor (*n* = 6). Statistical analysis was performed by using (A–C and E) Mann-Whitney test and (K) Wilcoxon matched-pairs signed rank test. ns: not significant, ∗*p* ≤ 0.05, ∗∗*p* ≤ 0.01.

To evaluate T cell binding to CORA_sh receptor variants, CORA_sh^+^ and CORA_sh_mod^+^ SPI-801 cells were co-cultured with CD8^+^ T cells isolated from HLA-A∗02-positive healthy individuals to analyze the T cell response upon recognition of foreign peptides presented in the HLA-A∗02 complex of the CORA receptor. These CD8^+^ T cells exhibited a significantly increased expression of CD25, CD69, and CD137 as markers for T cell activation and T cell proliferation, as well as slightly increased release of cytotoxic mediators (e.g., interferon [IFN]-γ) following co-culture with CORA_sh^+^ SPI-801 (Figures 4A–4I). In contrast, the same CD8^+^ T cells were neither activated nor proliferated when co-cultured with CORA_sh_mod^+^ SPI-801 cells, indicating that recognition of the CORA_sh_mod receptor by CD8^+^ T cells was prevented by the modification. Additionally, to evaluate expansion of peptide-specific T cells, CD8^+^ T cells isolated from HLA-A∗02-positive healthy individuals were co-cultured with the same CORA_sh^+^ or CORA_sh_mod^+^ SPI-801 cells that were previously loaded with the HLA-A∗02-restricted CMV pp65-derived NLV peptide. T cells specific for the HLA-A∗02/pp65_NLV_ complex specifically expanded to a frequency of 3.9% following co-culture with pp65_NLV_-loaded CORA_sh SPI-801 cells compared with a frequency of 0.7% following co-culture with unloaded CORA_sh SPI-801 cells as detected by multimer staining in flow cytometry (Figures 4J and 4K). In contrast, co-cultivation with pp65_NLV_-loaded CORA_sh_mod^+^ SPI-801 did not increase the frequency of HLA-A∗02/pp65_NLV_-specific T cells, indicating an impaired binding to the modified CORA receptor/pp65_NLV_ complex by T cells. In line, upon co-cultivation of HLA-A∗02/pp65_NLV_-specific T cells enriched from HLA-A∗02-positive healthy individuals with the same transduced and peptide-loaded SPI-801, they only reduced viability of pp65_NLV_-loaded CORA_sh^+^ SPI-801, but not pp65_NLV_-loaded CORA_sh_mod^+^ SPI-801, when compared with co-cultures with corresponding unloaded control cells (Figure 4L).

Thus, application of CORA_sh_mod-Ts is expected to preclude sensitization of T cells of potential transplant and CORA-T-cell therapy recipients toward foreign components of the HLA/peptide complex of the CORA receptor.

### CORA_sh- and CORA_sh_mod-Ts exhibit effective and target-specific T cell signaling, activation, and cytokine release

**Figure 5:**
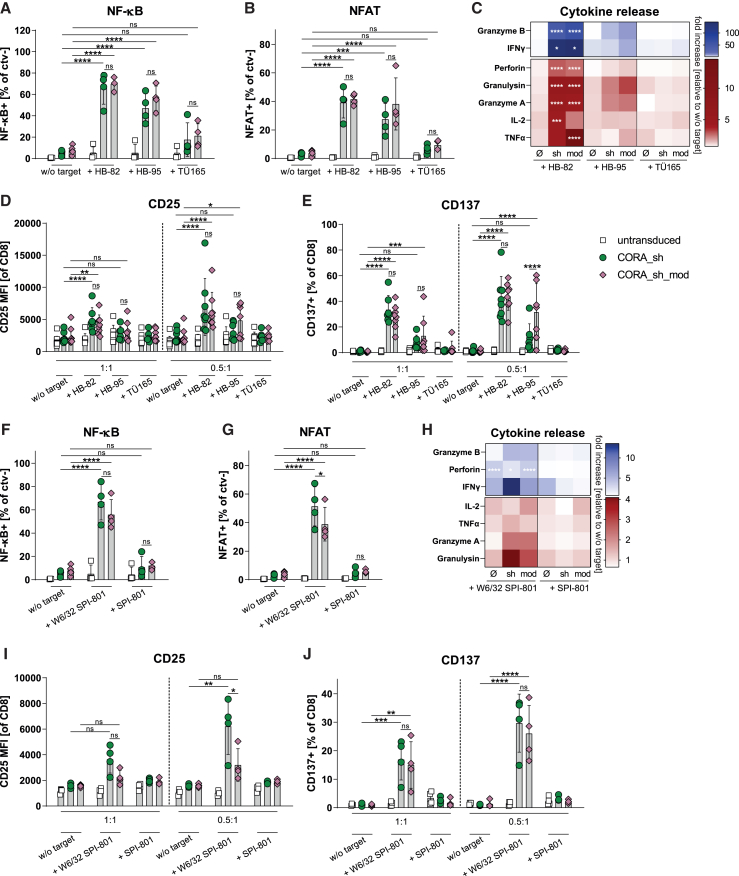
CORA_sh- and CORA_sh_mod-Ts exhibit effective and target-specific T cell signaling, activation, and cytokine release CORA_sh and CORA_sh_mod receptors were transduced into (A, B, F, G) Jurkat-based reporter cells or (C–E and H–J) primary CD8^+^ T cells. Respective untransduced cells served as controls. (A, B, F, and G) After cultivation of transduced reporter cells without (w/o) target cells or with the indicated target cells in an E:T ratio of 1:1 for 24h (A and F) NF-κB-induced eCFP or (B and G) NFAT-induced eGFP reporter expression was evaluated by flow cytometry (*n* = 4). (C and H) Transduced and untransduced (ø) T cells were co-cultured with the indicated target cells in an E:T ratio of 1:1 for 48 h. Release of soluble mediators into the supernatant was assessed by LEGENDplex. Fold increase to respective T cell cultures w/o target is shown as mean (*n* = 4–8). (D, E, I, and J) CORA-Ts were co-cultured with target cells in the indicated E:T ratios for 48 h. Expression of activation markers was evaluated by flow cytometry. Data are shown as scattered dot plot with mean ± SD, whereby each symbol represents an independent donor (*n* = 4–10). (A–J) Statistical analysis was performed by using two-way ANOVA with Tukey’s multiple comparisons test. ns: not significant, ∗*p* ≤ 0.05, ∗∗*p* ≤ 0.01, ∗∗∗*p* ≤ 0.001, ∗∗∗∗*p* ≤ 0.0001.

Both the CORA_sh- and the CORA_sh_mod receptor were then evaluated for specificity and effector functionality in detail using target-positive and -negative hybridoma cell lines. In the reporter assay, significant activation of NF-κB and NFAT was induced by CORA_sh and CORA_sh_mod receptors following target recognition on anti-HLA-A∗02 HB-82 and on anti-HLA-A/B/C HB-95 cells (Figures 5A and 5B). In that, CORA_sh_mod^+^ reporter cells increased transcription factor activation in the same extent as CORA_sh^+^ reporter cells. Importantly NF-κB and NFAT were not activated following co-culture with target-negative TÜ165 hybridoma cells, indicating specificity of both CORA receptors.

The CORA receptors were then transduced into primary CD8^+^ T cells. CORA_sh- and CORA_sh_mod-Ts generated from HLA-A∗02-negative or -positive CD8^+^ T cells as starting material could be expanded with similar cell counts, viability, activation levels, and cytokine release compared with corresponding untransduced T cells (Figures S5 and S6). After their generation and enrichment via EGFRt, co-cultures with autologous PBMCs moreover revealed that neither CORA_sh nor CORA_sh_mod-Ts did mediate cytotoxicity toward immune cells within PBMCs (Figure S7), so that fratricide and unintended targeting of other immune cells could be excluded.

CORA_sh-Ts and CORA_sh_mod-Ts exhibited expression of HLA-A∗02 complex on 76%–85% of cells assessed by either anti-HLA-A∗02 antibody or HB-82 supernatant staining in flow cytometry (Figures S8A–S8F). However, the MFIs of both antibody stainings tended to be lower for CORA_sh_mod-Ts (2800 and 2900) when compared with CORA_sh-Ts (3400 and 4400) indicating a slightly, but not significantly reduced receptor expression per cell (Figures S8C and S8F). In line with the target-specific induction of transcription factor activation (Figures 5A and 5B), primary CORA_sh- and CORA_sh_mod-Ts induced expression and release of effector molecules and pro-inflammatory cytokines (e.g., granzyme B, IFN-γ, perforin, and granulysin) and were activated (indicated by upregulation of CD25, CD137) following co-culture with HB-82 and HB-95 cells, but not with TÜ165 cells (Figures 5C–5E, S8G, and S8H). Interestingly, despite similar frequencies and MFIs of BCRs on HB-82 and HB-95 cells (Figures 2B and S2A), CORA_sh- and CORA_sh_mod-Ts exhibited an increased cytokine release and activation toward HB-82 when compared with HB-95 cells. To decipher reactivity of CORA-Ts toward W6/32 receptors, which is the specificity of BCRs on HB-95 cells, in detail, we utilized SPI-801 cells transduced with a membrane-bound W6/32 single chain variable fragment (scFv; W6/32 SPI-801) as additional target-positive cells and respective untransduced SPI-801 cells as negative controls. Upon specific recognition of expressed W6/32 scFv, transcription factor activity, release of effector molecules and expression of CD25 and CD137 were induced by CORA_sh- and CORA_sh_mod-Ts in a significant and comparable extent, whereby both CORA-T products did not react toward untransduced SPI-801 cells (Figures 5F–5J).

Taken together, CORA_sh- and CORA_sh_mod-Ts exhibit distinct target specificity for anti-HLA-A∗02 target cells and specifically induce multiple effector functions, whereby the amino acid substitutions for abrogation of T cell sensitization in the CORA_sh_mod receptor did not influence target functionality.

### CORA_sh- and CORA_sh_mod-Ts mediate target-specific cytotoxicity resulting in effective reduction of anti-HLA antibody release

**Figure 6:**
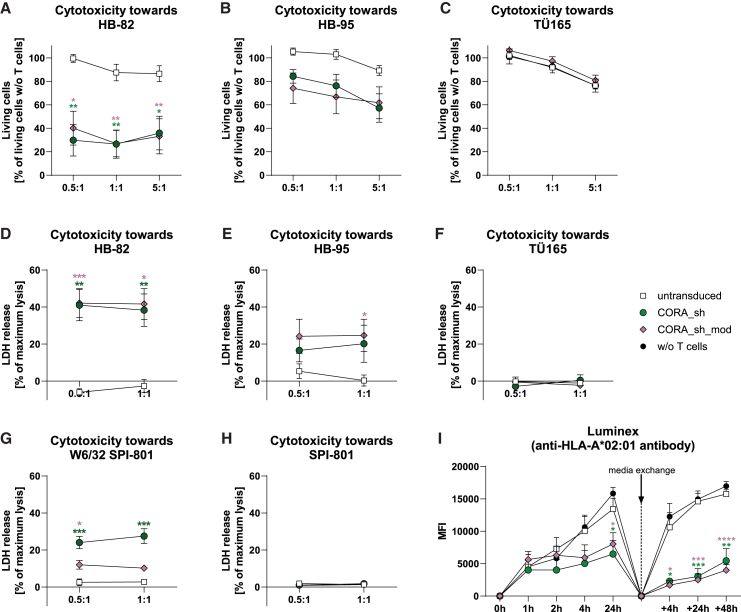
CORA_sh- and CORA_sh_mod-Ts mediate target-specific cytotoxicity resulting in effective reduction of anti-HLA antibody release (A–H) CORA_sh- and CORA_sh_mod-Ts were co-cultured with indicated target cells in the indicated E:T ratios for 48 h. Respective co-cultures with untransduced T cells served as controls. (A–C) Prior to the co-culture, target cells were labeled with CTV to determine the frequency of living target cells (CTV^+^7-AAD^−^) after co-culture (*n* = 6–7). Frequencies of living target cells in co-cultures with indicated T cells were then normalized to corresponding frequencies of viable target cells in cultures without (w/o) T cells. (D–H) Co-culture supernatants were evaluated for LDH levels as indicator for T cell-mediated cytotoxicity (*n* = 4–7). (I) Generated CORA-Ts were co-cultured with HB-82 cells in an E:T ratio of 5:1. At the indicated time points, supernatants were analyzed for released anti-HLA-A∗02 antibody by Luminex. After 24 h, co-culture media were replaced by fresh medium, after which further measurements were performed at indicated time points (“+”). Data are shown as mean + SEM (*n* = 5). Significances are shown in comparison with HB-82 cultured without (w/o) T cells and evaluated at the respective same time points. (A–H) Data are shown as mean ± SEM. Statistical analysis was performed by using two-way ANOVA with Dunnett’s multiple comparisons test. Significances are shown in comparison with respective untransduced T cells cultured in the same E:T ratio. ∗*p* ≤ 0.05, ∗∗*p* ≤ 0.01, ∗∗∗*p* ≤ 0.001, ∗∗∗∗*p* ≤ 0.0001.

The cytotoxic capacity of CORA_sh- and CORA_sh_mod-Ts toward anti-HLA target cells was evaluated by co-cultivation with hybridoma cells for 48 h, followed by either analysis of living (7AAD^−^) target cells via flow cytometry or LDH assay using the co-culture supernatant. In these assays, both CORA-Ts significantly reduced viability of HB-82 and HB-95 cells, whereby CORA_sh- and CORA_sh_mod-Ts exhibited equal cytotoxic capacity (Figures 6A–6F). The elimination of HB-82 was higher when compared with HB-95 cells. In contrast, in line with a lack of T cell activation and cytokine production, CORA_sh- and CORA_sh_mod-Ts did not mediate cytotoxicity toward TÜ165 cells, indicating their specificity for anti-HLA-A∗02 BCRs. To confirm the receptor-specific functionality, W6/32 SPI-801 and corresponding untransduced SPI-801 cells were used as additional target cells (Figures 6G and 6H). The LDH release into the co-culture supernatant as indicator for target-cell death was significantly increased in co-cultures of CORA_sh- or CORA_sh_mod-Ts with W6/32 SPI-801 cells when compared with co-cultures of untransduced T cells with W6/32 SPI-801 cells. In that, CORA_sh-Ts showed a slightly increased cytotoxic capacity when compared with CORA_sh_mod-Ts. Neither of the CORA-T products mediated elimination of untransduced SPI-801 cells confirming the W6/32 scFv as recognized target molecule.

Importantly, elimination of HB-82 cells by CORA_sh- and CORA_sh_mod-Ts lead to a significantly reduced release of anti-HLA-A∗02 antibody into the co-culture supernatant as assessed by Luminex (Figure 6I). This was especially profound following an exchange of culture media after 24 h, after which anti-HLA-A∗02 concentrations in supernatants of HB-82 cultured alone or together with untransduced T cells quickly increased within hours of cultivation, whereas the increase was significantly reduced in co-cultures of HB-82 cells with either CORA_sh- or CORA_sh_mod-Ts.

Thus, CORA_sh- and CORA_sh_mod-Ts specifically and efficiently eliminate anti-HLA-A∗02 target cells resulting in an abrogation of anti-HLA-A∗02 antibody release by these cells.

### CORA_sh_mod-Ts significantly reduce anti-HLA B-cell growth *in vivo*

**Figure 7:**
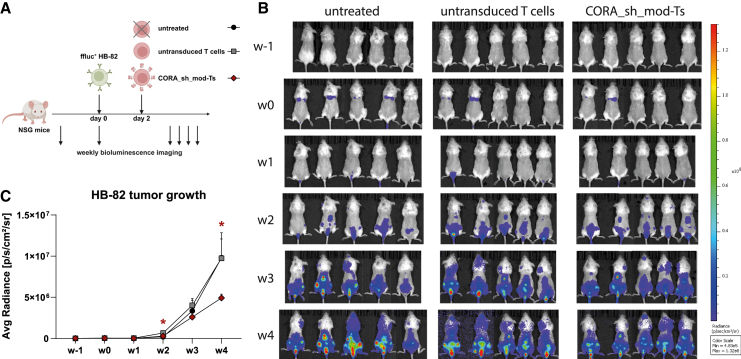
CORA_sh_mod-Ts significantly reduce anti-HLA B-cell growth *in vivo* (A) Female NSG mice were injected 5 × 10^5^ ffluc^+^ HB-82 cells followed by injection of 5 × 10^5^ CORA_sh_mod-Ts or untransduced T cells 2 days later. Respective mice injected with ffluc^+^ HB-82 cells but not treated with T cells (untreated) served as controls. CORA_sh_mod-Ts were generated by transduction of CD4^+^ and CD8^+^ T cells and expansion using a protocol similar to manufacturing of CAR-Ts on the CliniMACS Prodigy. (B and C) Bioluminescence imaging was performed every week (w) to assess growth of HB-82 cells. Images in w0 were taken 15–20 min after injection of ffluc^+^ HB-82 cells. (C) Quantification of bioluminescence was performed by setting a defined square area set on each animal to obtain average (Avg) radiance values. Data are shown as mean + SEM (*n* = 5). Statistical analysis was performed by using Mann-Whitney test. Significances are shown in comparison with respective mice treated with untransduced T cells at the same time points. ∗*p* ≤ 0.05.

The cytotoxic capacity of CORA_sh_mod-Ts toward anti-HLA target cells was further assessed in an HB-82 NOD.Cg-*Prkdc*^*scid*^ *Il2rg*^*tm1Wjl*^/SzJ (NSG) mouse model (Figure 7A). To quantify target-cell burden by bioluminescence measurements, HB-82 cells were transduced to stably express firefly luciferase (ffluc). Mice were injected with 5 × 10^5^ ffluc^+^ HB-82 cells, followed by injection of 5 × 10^5^ CORA_sh_mod-Ts on day 2. CORA_sh_mod-Ts for these experiments were manufactured using CD4^+^ and CD8^+^ T cells as starting population and a protocol similar to the manufacturing of CAR-Ts at the CliniMACS Prodigy^11^ for improved persistence and comparability with cells manufactured for clinical application. *In vitro*, manufacturing of these CD4^+^/CD8^+^ CORA_sh_mod-Ts yielded similar cell numbers, comparably low exhaustion, and even a lower proportion of terminally differentiated cells following expansion when compared with respective CD8^+^ CORA_sh_mod-Ts generated by using the previous expansion protocol (Figures S9A–S9C). Moreover, while cytotoxicity and target specificity toward HB-82 cells, as well as intracellular pro-inflammatory cytokine expression were similar to CD8^+^ CORA-Ts, CD4^+^/CD8^+^ CORA-Ts exhibited a significantly increased T cell activation following target recognition (Figures S9D–S9H), indicating a supporting role of CD4^+^ T cells that was described to mediate superior antitumor reactivity *in vivo*.^12^

Using the HB-82 mouse model, bioluminescence measurements following administration of the ffluc substrate D-luciferin revealed rapid cell growth of ffluc^+^ HB-82 cells, mainly located in the bone marrow, in untreated mice or mice treated with untransduced T cells (Figures 7B and 7C). Although treatment of mice with CORA_sh_mod-Ts did not prevent appearance and distribution of ffluc^+^ HB-82 cells, growth of these cells was significantly reduced in mice treated with CORA_sh_mod-Ts in comparison to respective mice treated with untransduced T cells. The reduced growth of HB-82 cells in mice treated with CORA_sh_mod-Ts did not translate into significantly lower anti-HLA-A∗02 concentrations measured by Luminex in blood samples of the mice (Figure S10), most likely due to the restricted duration of the experiment, which was terminated after 4 weeks.

Thus, while CORA_sh_mod-Ts did not mediate clearance of HB-82 cells *in vivo*, most likely due to the aggressive nature of the HB-82 (tumor) model that restricted evaluation of the reduction of anti-HLA antibody levels in the long-term, a significant reduction of anti-HLA B-cell growth by CORA_sh_mod-Ts could be confirmed *in vivo*.

### CORA-Ts mediate specific elimination of HLA-specific B cells in target-cell mixtures and can be modified to resist tacrolimus treatment

**Figure 8:**
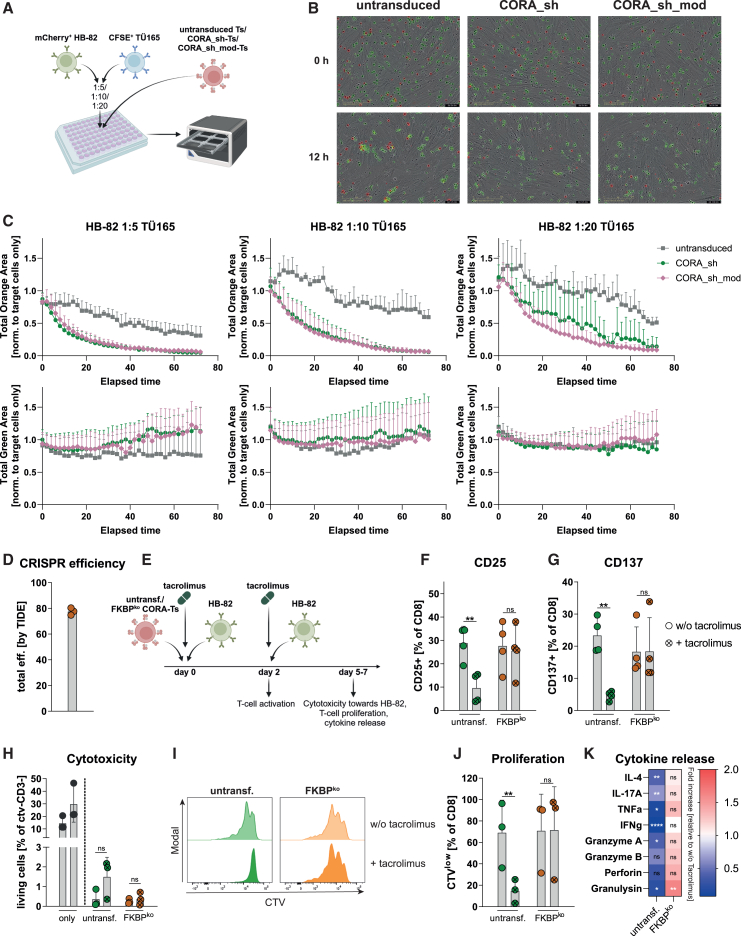
CORA-Ts mediate specific elimination of HLA-specific B cells in target-cell mixtures and can be modified to resist tacrolimus treatment (A) CORA_sh- and CORA_sh_mod-Ts were co-cultured in a 1:1 ratio with target-cell mixtures composed of mCherry^+^ HB-82 with CFSE^+^ TÜ165 cells in the indicated ratios. Respective co-cultures with untransduced T cells served as controls. Live-cell imaging using the Incucyte Live-Cell Analysis System (Sartorius) was performed to evaluate specific target-cell elimination. (B) Representative pictures of selected time points of co-cultures with target-cell mixtures in a 1:5 ratio. (C) Total orange or green object areas were analyzed by using the Incucyte software (Sartorius) and normalized to respective values of target-cell mixtures cultured without T cells (target cells only) for every time point. Data are shown as mean ± SD of two independent experiments with two donors each. In one experiment, HFF cells were seeded on all wells as scaffold 1 day before setting the triple co-cultures. (D–K) CORA_sh receptors were transduced into CD8^+^ T cells, followed by transfection with FKBP12-targeting RNP complex (FKBP^ko^). Respective untransfected CORA_sh-Ts served as controls (untransf.). (D) CRISPR efficiency was assessed by analysis of sequencing results by TIDE. (E–G) CORA-Ts were co-cultured with HB-82 cells for in an E:T ratio of 1:1 for 48 h in presence or absence (w/o) of 5 ng/mL tacrolimus, after which expression of activation markers was evaluated by flow cytometry (*n* = 4). (E and H–K) Prior to the co-culture, CORA-Ts were labeled with CTV. After 2 days of co-culture, CORA-Ts were re-stimulated with the same number of HB-82 cells (*n* = 3). After 5–7 days of co-culture, (H) the frequency of living target cells (CTV^−^CD3^−^7-AAD^−^) was assessed by flow cytometry. HB-82 cells cultured alone (only) for 5–7 or 3–5 days, respectively, are shown as first and second bar. (I and J) Proliferation of CORA-Ts was assessed via CTV dilution assay by flow cytometry and is shown as (I) representative histograms or (J) mean. (K) Release of soluble mediators into the supernatant was assessed by LEGENDplex. Fold increase to respective co-cultures in absence of tacrolimus is shown as mean. Statistical analysis was performed by using two-way ANOVA with Šídák’s multiple comparisons test. (D, F–H, and J) Data are shown as scattered dot plot with mean ± SD, whereby each symbol represents an independent donor. Statistical analysis was performed by using two-way ANOVA with Tukey’s multiple comparisons test. ns: not significant, ∗*p* ≤ 0.05, ∗∗*p* ≤ 0.01, ∗∗∗∗*p* ≤ 0.0001.

As a more realistic model representing a lower number of alloreactive, anti-HLA B-cell clones among B cells with other specificities, triple co-cultures containing CORA-Ts and target-cell mixtures of HB-82 and TÜ165 were performed (Figures 8A–8C). For that, HB-82 cells were transduced to express mCherry and mixed with the control TÜ165 cells labeled with carboxyfluorescein succinimidyl ester (CFSE) in decreasing ratios. Following addition of unlabeled CORA-Ts, elimination of respective target-positive and -negative cells was assessed in real-time measurements using live-cell imaging. In all triple co-cultures, CORA_sh-Ts and CORA_sh_mod-Ts rapidly eliminated mCherry^+^ HB-82 cells (Figures 8B and 8C). Importantly, the growth of CFSE^+^ TÜ165 cells was not reduced by both CORA-Ts in the same triple co-cultures demonstrating their specificity even in target-cell mixtures of B cells with different specificities.

As therapy to prevent organ rejection, most SOT patients are continuously administered immunosuppressive medication, e.g., the calcineurin inhibitor tacrolimus. To overcome a reduction of CORA-T functionality under immunosuppressive conditions, they were endowed with resistance toward tacrolimus treatment as previously demonstrated for CMV-specific T cells.^13^ For that, besides transduction with the CORA_sh receptor, a CRISPR-Cas9-mediated knockout of the tacrolimus-binding protein gene *FKBP12* achieving CRISPR efficiencies of 80% was implemented in the CORA-T manufacturing process (Figure 8D). Functionality of generated tacrolimus-resistant FKBP^ko^-CORA_sh-Ts in comparison with unmodified CORA_sh-Ts was tested by co-cultivation with HB-82 cells with or without addition of tacrolimus in a clinically relevant dose of 5 ng/mL^14^ (Figure 8E). Upregulation of CD25 and CD137 on CORA_sh-Ts following co-culture with HB-82 cells as markers for T cell activation was significantly reduced in the presence of tacrolimus when compared with respective co-cultures without tacrolimus addition (Figures 8F and 8G). In contrast, activation of FKBP^ko^-CORA_sh-Ts following recognition of HB-82 cells was unaffected by the presence of tacrolimus. Cytotoxicity of CORA_sh-Ts toward HB-82 cells was not reduced after co-cultivation for 48 h in the presence of tacrolimus; however, their cytotoxic capacity toward newly added HB-82 cells after re-stimulation was slightly reduced in presence of tacrolimus (Figure 8H). In contrast, FKBP^ko^-CORA_sh-Ts mediated equally high cytotoxicity irrespective of immunosuppressive treatment. In the same re-stimulation co-culture with HB-82 cells, T cell proliferation measurements by CTV dilution assay revealed a significantly diminished proliferation of CORA_sh- but not of FKBP^ko^-CORA_sh-Ts in presence of tacrolimus (Figures 8I and 8J). In line, the release of several pro-inflammatory cytokines and effector molecules (e.g., TNF-α, IFN-γ and granulysin) in response to HB-82 recognition was significantly reduced by tacrolimus addition to CORA_sh-T, but not to FKBP^ko^-CORA_sh-T co-cultures (Figure 8K).

Thus, as next steps toward predicting elimination of alloreactive B cells in transplant patients, CORA-Ts were shown to mediate specific elimination of HLA-A∗02-specific B cells in mixtures with B cells of different specificities, and could be modified by CRISPR-Cas9-mediated knockout of the *FKBP12* gene to maintain potent effector functions irrespective of the presence of tacrolimus.

Our results demonstrate that, by engineering of T cells with an innovative CORA receptor, they are able to specifically recognize and eliminate distinct anti-HLA B cells, having the potential to selectively prevent the formation of anti-HLA antibodies even under immunosuppressive conditions. This suggests CORA-Ts as a potent novel approach to specifically eliminate alloreactive anti-donor-HLA B cells to combat AMR and to improve long-term graft survival in SOT patients while preserving their overall B cell immunity.

## Discussion

In the present study, we developed a cell therapeutic approach to specifically eliminate anti-donor-HLA B cells and thereby abrogate release of anti-HLA DSAs responsible for AMR, while preserving B cells with different specificities. To redirect autologous T cells against distinct anti-HLA B cells, we designed a CORA receptor that is composed of an HLA molecule of interest fused to 4-1BB and CD3ζ T cell signaling domains commonly used for CARs.^15^ As proof-of-concept, a truncated HLA-A∗02 α-chain was encoded in the CORA receptor and shown to be assembled with β_2_m and peptides upon transduction into cells by the endogenous machinery, whereby a short spacer domain in the receptor enabled superior HLA-complex expression on all transduced cells when compared with a long spacer domain variant. In line, recognition of anti-HLA-A∗02 BCRs on the surface of HB-82 cells (anti-HLA-A∗02), and effector functionality including cytotoxicity toward the B cell line was improved for CORA_sh-Ts when compared with CORA_lo-Ts, matching to studies in CAR-Ts that described a correlation between effector functionality and density of CAR expression.^16^

CORA_sh-Ts were moreover shown to be highly specific by utilizing additional hybridoma cells and artificial target cells with W6/32 specificity, revealing potent and anti-HLA-A∗02-specific induction of T cell signaling and activation, release of cytokines and effector molecules, as well as cytotoxicity. As main aim in the approach to combat AMR, they moreover efficiently reduced the release of anti-HLA-A∗02 antibodies. Effector functionality of CORA-Ts was higher toward HB-82 than toward HB-95 cells, likely due to lower frequency of HB-95 cells that could be stained with eukaryotic HLA-A∗02 multimer when compared with HB-82 cells, indicating that CORA-T functionality is specific to availability of HLA-specific BCRs. In line, CORA_sh-Ts did not react toward target-negative cells including the anti-HLA-B∗35 B cell line TÜ165. Specificity of CORA-Ts was confirmed in live-cell imaging experiments utilizing triple co-cultures with target-cell mixtures modeling the situation of low numbers of alloreactive B cells among B cells with various specificities in transplant patients. Thus, as opposed to currently available B cell depletion or immunosuppression protocols,^3^ CORA-T cell therapy would potentially preserve the general humoral immunity for defense against pathogens and maintain supportive B_reg_ subpopulations beneficial for allograft tolerance.

The CORA_sh receptor was further modified by the exchange of two amino acids (D227K and T228A) in the HLA-A∗02 α_3_ domain. These amino acids are known to be part of a negatively charged α_3_-chain loop important for binding of CD8.^17^ The amino acid exchange did not affect the overall functionality of CORA-Ts. Purbhoo et al. could show that the utilized substitutions in HLA-A∗02 multimers were sufficient to efficiently reduce CD8^+^ T cell activation following multimer staining.^10^ In line, in the present study, the CORA_sh_mod receptor, in contrast to the unmodified CORA_sh receptor, did not induce activation, proliferation, cytotoxic mediator release, or cytotoxicity of CD8^+^ T cells specific for HLA-A∗02 in complex with foreign, SPI-801-derived peptides or loaded with a distinct CMV pp65-derived peptide, most likely due to the described abrogation of CD8 binding. Thus, CORA_sh_mod-Ts are not expected to interact with alloreactive anti-donor-HLA T cells in the patient, which increases safety of the approach.

CORA_sh_mod-Ts significantly reduced the growth of ffluc^+^ HB-82 cells in an *in vivo* mouse model when compared with corresponding mice treated with untransduced T cells. Lack of complete HB-82 clearance and subsequent reduction of anti-HLA levels in the mice by CORA-Ts, most likely, could be attributed to the aggressive HB-82 (tumor) model that required termination of the experiment after 4 weeks, and the low number of applied CORA-Ts in comparison with HB-82 cells. Whereas the utilized ratio of T cells to HB-82 cells would be suitable to evaluate the treatment of a solid tumor, the number of alloreactive, anti-HLA B cell clones following SOT among B cells with various specificities is expected to be significantly lower. Using HLA-A∗02 tetramer staining, Zachary et al. found 4.1% of tetramer^+^ B cells in presensitized end-stage renal disease patients, and 1.6% in nonsensitized patients.^18^ Thus, evaluation of CORA-Ts in a syngeneic mouse model of immunocompetent, HLA-sensitized mice, optimally even in a transplant model, would be more suitable and required to confirm their efficacy to eliminate alloreactive B cells while preserving the overall immune function in a future study.

As further modification, CRISPR-Cas9-mediated knockout of the corresponding binding protein gene *FKBP12* endowed CORA-Ts with potent protection from tacrolimus treatment. Calcineurin inhibitors, such as tacrolimus, are currently the most effective and therefore a widely used class of maintenance immunosuppression following SOT, since they potently reduce the activation, maturation, and cytokine secretion by T cells.^13^ Thus, tacrolimus-resistant CORA-Ts are a promising approach with expected high functionality even under immunosuppressive conditions in transplanted patients.

The CORA-T strategy^19^ clearly surpasses the concurrently described chimeric HLA antibody receptor (CHAR) T cells^20^^,^^21^ in several key aspects. The superiority of CORA-Ts is evident through the incorporation of crucial modifications designed to (1) prevent sensitization of alloreactive T cells and (2) provide drug resistance, ensuring their efficacy in eliminating anti-HLA B cells even under immunosuppressive conditions. These enhancements are pivotal for clinical application, especially in the treatment of SOT patients. Notably, (1) TMR mediated by alloreactive T cells following AMR remains the second leading cause of late allograft failure,^2^ and (2) transplanted patients typically require ongoing immunosuppression. Thus, the CORA-T strategy offers a significantly more effective solution than CHAR T cells.

As potential drawback of CORA-Ts as a new therapy approach, whereas alloreactive naive and memory B cells are characterized by anti-HLA BCRs, plasma cells express little or no surface BCR. Thus, equal to current B cell depletion protocols, not all plasma cells are expected to be efficiently eliminated. However, recently CD19-CAR-Ts were proven to be highly efficient in the treatment of autoimmune diseases, including systemic lupus erythematosus, idiopathic inflammatory myositis, and systemic sclerosis, by elimination of autoantibody-releasing B cells, although they were shown not to deplete long-lived plasma cells.^22^^,^^23^ Otherwise, to also target plasma cells, a promising approach could be the combination of CORA-Ts with proteasome inhibition, e.g., by bortezomib, to induce plasma cell apoptosis. In a randomized controlled trial among kidney recipients with AMR, bortezomib treatment alone could not prevent progression of AMR,^24^ which was attributed to an only transient decrease in DSA-producing cells followed by an immediate increase in germinal center B cells, again reconstituting memory and plasma B cells.^25^ Thus, combination with CORA-T cell treatment could potentially prevent reconstitution of anti-HLA plasma cells. However, all plasma cells including protective virus-specific cells would be depleted by bortezomib treatment. Otherwise, pre-treating nonsensitized patients on the waiting list with CORA-Ts to prevent development of AMR by depletion of naive HLA-specific B cells would circumvent formation of corresponding plasma cells while retaining the general B cell immunity. As additional combination, plasmapheresis could prevent potential masking and reduction in functionality of CORA-Ts by DSAs present in the blood of transplant patients. However, this is contradicted by data from a cell therapeutic approach targeting autoreactive B cells via their BCRs in the context of pemphigus vulgaris (PV).^26^ Here, soluble autoantibodies interacting with the engineered T cell receptor were shown to induce low-level proliferation of the engineered T cells expected to increase their efficacy and persistence.^27^ Functionality of these chimeric autoantigen receptor T cells (CAAR-Ts) could be impressively demonstrated using different *in vitro* and *in vivo* models, leading to their evaluation in an ongoing, currently recruiting clinical study (NCT04422912). Suitability of T cell therapy approaches to target B cell populations via their BCRs could moreover be confirmed by preclinical development of so-called B cell antibody receptor (BAR)-engineered T cells as a tool against anti-factor VIII antibody-releasing B cells in hemophilia A patients,^28^ as well as with CAAR-Ts targeting autoantigen-specific B cells in muscle-specific tyrosine kinase myasthenia gravis^29^ and NMDA receptor encephalitis.^30^

In line, we here present a promising new tool to selectively eliminate alloreactive anti-HLA B cells responsible for AMR even under immunosuppressive conditions while preserving the protective B cell immunity. By using CORA-Ts targeting anti-HLA-A∗02 B cells as a proof-of-concept, future development of CORA-Ts based on further HLA class I and class II alleles will broaden the applicability to a larger cohort of transplant patients in an individual and patient-tailored manner.

## Materials and methods

### Human sample materials

All experiments with primary cells were performed using residual blood samples from routine platelet collection at the Institute of Transfusion Medicine and Transplant Engineering, Hannover Medical School, for which written informed consent was obtained from all donors as approved by the local ethics committee (2519–2014, 3639–2017). Residual frozen sera samples from HLA-sensitized patients were evaluated, which was approved by the Ethics Committee of Hannover Medical School (8969_BO_K_2020).

### Construction of CORA receptors

To design the CORA receptors, sequences of HLA-A∗02:01:01:01 exons 1–4 (GenBank no. HG794376.1) were synthesized (Thermo Fisher Scientific, Waltham, MA, USA) and cloned into two previously described TÜ165-CAR-epHIV7 vectors with either a short 12-aa or long 229-aa spacer domain to, respectively, replace the signaling peptide and TÜ165 scFv.^31^

To generate CORA receptors modified to abrogate T cell binding to the HLA-A∗02 domain (CORA_sh_mod and CORA_lo_mod), two residues (D227K and T228A) in the α_3_ domain of HLA-A∗02:01 described to abrogate binding of CD8^10^ were changed by site-directed mutagenesis (Agilent, Santa Clara, CA, USA). Further details as well as generation and titration of lentiviral vectors are provided in the Supplemental Methods.

### Detection of anti-HLA-A∗02 antibodies by multi-analyte flow array

Serum of kidney transplant recipients was evaluated for presence of anti-HLA-A∗02:01 antibodies using mixed HLA antigen-charged polysterene beads (LIFECODES LifeScreen LSA test, Immucor, Norcross, GA, USA) and a multi-analyte flow array (Luminex 200 System, Thermo Fisher Scientific) according to the manufacturer’s instructions. Murine anti-HLA-A∗02:01 antibodies released by HB-82 cells were analogously evaluated, except for the usage of an anti-mouse immunoglobulin (Ig)G (H + L) polyclonal F(ab’)_2_ fragment (Jackson Immunoresearch, West Grove, PA, USA) for detection.

### Evaluation of anti-HLA antibody specificity by crossmatch

PBMCs or CORA receptor transduced SPI-801 cells (ACC 86; DSMZ, Braunschweig, Germany) were incubated with serum of HLA-sensitized patients or cell culture supernatants of hybridoma cells, followed by addition of rabbit complement (Bio-Rad, Hercules, CA, USA). FluoroQuench Stain/Quench reagent (Thermo Fisher Scientific) was used to distinguish live cells (green) from dead cells (red) by using a BZ-8100E microscope (Keyence, Osaka, Japan). Crossmatch scores were determined by using values 1 (≤10%), 2 (10%–20%), 3 (20%–40%), 6 (40%–80%), and 8 (80%–100%) for increasing frequencies of dead cells according to standard protocols of the National Institutes of Health (USA).^8^

### Reporter assay to determine CORA receptor signaling

CORA receptors were transduced into a previously described Jurkat-based reporter cell line^9^ by addition of lentiviral particles in a multiplicities of infections (MOIs) of 1 and 5 μg/mL Polybrene Infection/Transfection Reagent (Merck, Darmstadt, Germany) using spinoculation. After 48 h, transduced reporter cells were co-cultured with the indicated target cells previously labeled with CellTrace violet proliferation dye (CTV; Thermo Fisher Scientific) for 24 h in an effector to target (E:T) ratio of 1:1. CORA receptor-induced signaling was assessed by detection of enhanced cyan fluorescent protein (eCFP) reporting for NF-κB and enhanced green fluorescent protein (eGFP) reporting for NFAT activation in CTV^−^ reporter cells by flow cytometry. Untransduced reporter cells were treated analogously and used as control.

### Generation of primary, tacrolimus-resistant, CORA-Ts

Primary CORA-Ts were generated as previously described.^31^ Briefly, peripheral blood mononuclear cells (PBMCs) were isolated from residual blood samples from routine platelet collection of healthy individuals by density gradient centrifugation using Lymphosep (c.c.pro, Oberdorla, Germany). CD8^+^ T cells were isolated using negative selection (Miltenyi Biotec, Bergisch Gladbach, Germany), activated with anti-CD3/CD28 beads (Thermo Fisher Scientific) in a ratio of 1:1, and cultured in TexMACS (Miltenyi Biotec) with 3% human serum (c.c.pro; T cell medium) supplemented with 12.5 ng/mL IL-7 and IL-15 (PeproTech, Cranbury, NJ, USA). On day 1, T cells were transduced with CORA receptors by addition of lentivirus in an MOI of 3, 5 μg/mL Polybrene Infection/Transfection Reagent, and spinoculation. On day 2, the anti-CD3/CD28 beads were removed.

For the generation of tacrolimus-resistant CORA-Ts, beads were removed on day 3 followed by electroporation using the Human T cell Nucleofector Kit (Lonza, Basel, Switzerland) and the Amaxa Nucleofactor 2b (Lonza, program T-023) to transfer the RNP complex of 62 pmol of Alt-R *Streptococcus pyogenes* Cas9 protein V3 precomplexed with 72 pmol duplex of FKBP12-targeting crRNA (5′-GGGCGCACCTTCCCCAAGCG-3′; sequence previously described^13^) and tracrRNA (all from Integrated DNA Technologies, Coralville, IA, USA).

CORA-Ts for the HB-82 mouse model were generated by using a small-scale protocol similar to the manufacturing of CAR-Ts at the CliniMACS Prodigy^11^: CD4^+^ and CD8^+^ T cells were isolated by combination of positive selection kits (Miltenyi Biotec), activated with T cell TransAct (Miltenyi Biotec), and then cultured and transduced with CORA_sh_mod receptors as described above. On day 2, cells were resuspended in fresh T cell medium and 12.5 ng/mL IL-7 and IL-15 to remove the T cell TransAct.

After that, all cells were split depending on their growth. On day 8–9, transduced T cells were enriched via co-expressed EGFRt. EGFRt^+^ cells were then further expanded until day 12–16. Untransduced T cells were expanded analogously, except for the addition of lentivirus and enrichment of EGFRt^+^ cells and served as control.

For evaluation of on-target editing efficiency of tacrolimus-resistant CORA-Ts, DNA was isolated on day 12–16 by using the DNeasy Blood and Tissue Kit (Qiagen, Hilden, Germany). The FKBP12 site was amplified with the Platinum SuperFi DNA Polymerase (Thermo Fisher Scientific) according to the manufacturer’s instructions and using previously described primers.^13^ PCR products were purified (Qiagen), Sanger sequenced (Microsynth Seqlab, Goettingen, Germany), and CRISPR efficiency was determined by TIDE software version 3.3.0.^32^

### Co-cultures for evaluation of CORA-T-cell functionality

CORA-T cell functionality was evaluated following co-culture with indicated target cells in T cell medium. Target-cell lines and their generation are described in the Supplemental Methods. For detection of anti-HLA-A∗02 antibodies, samples of co-culture supernatants were taken at the indicated time points, whereby media was replaced with new T cell medium after 24 h.

For evaluation of tacrolimus-resistant CORA-Ts, they were co-cultured with HB-82 cells in absence or presence of 5 ng/mL tacrolimus (Merck) in an E:T ratio of 1:1 for 48 h. Cytotoxicity, proliferation, and cytokine release were assessed after 5–7 days, whereby T cells were re-stimulated with the same number of HB-82 cells after 2 days.

Antibodies used for flow cytometry are listed in Table S1 and further information on staining procedures is described in the Supplemental Methods.

### Measurement of effector molecule release by CORA-Ts using multiplex cytokine analysis

To determine effector molecule concentrations in supernatants, the LEGENDplex human CD8/NK Panel (BioLegend, San Diego, CA, USA) was performed according to the manufacturer’s instructions. Data were analyzed with LEGENDplex v8.0 software (BioLegend). Fold increase was calculated by division by concentrations of the respective CORA-Ts cultured alone.

### Evaluation of cytotoxic capacity of CORA-Ts

Killing capacity of CORA-Ts was assessed by co-cultivation with target cells for 48 h. Prior to the co-culture, either CORA-Ts or target cells were labeled with CTV. Afterward, 7-AAD (BioLegend or BD, Franklin Lakes, NJ, USA) was used to discriminate dead target cells via flow cytometry. As an alternative, cytotoxicity was determined based on the release of lactate dehydrogenase (LDH) into the co-culture supernatant measured by a Cytotoxicity Detection Kit (Roche, Basel Switzerland) and using a Synergy 2 Multi-Mode Microplate Reader (Biotek, Winooski, VT, USA). Maximum lysis was measured by addition of 1% Triton X-100 (Merck) to control wells. LDH release by eliminated target cells in co-cultures with CORA-Ts was calculated from absorbance values assessed in co-cultures corrected for values obtained from respective effector and target cells cultured alone and normalized to maximum lysis according to the manufacturer’s instructions using Equation 1.

(Equation 1) $$\text{LDH}\;\text{release}\;\left(\%\right)=\frac{\text{coculture}-\text{effector}\;\text{cell}\;\text{control}-\text{target}\;\text{cell}\;\text{control}}{\text{maximum}\;{\text{lysis}}_{\text{coculture}}-\text{target}\;\text{cell}\;\text{control}}\times 100$$

### Cytotoxicity measurements using live-cell imaging

Killing capacity of CORA-Ts toward target cells in mixtures of hybridoma cells with different specificities and elimination of CORA receptor-transduced SPI-801 cells was measured using live-cell imaging with the Incucyte SX5 or SX1 Live-Cell Analysis System (Sartorius, Goettingen, Germany). For that, respective target cells were transduced to express mCherry (lentivirus kindly provided by Axel Schambach, Christopher Baum and Michael Morgan, MHH, Hannover) or labeled with CFSE (Thermo Fisher Scientific) before. Unlabeled T cells were added and the measurement started following their settlement after 15 to 20 min. All conditions were set in duplicates and four pictures were taken per well every 2 h using a 20× objective. Total orange, green, or red object areas were analyzed by using the Incucyte 2022B and 2023B software (Sartorious). Obtained values were normalized as indicated in corresponding figure legends.

### HB-82 NSG mouse model

All mice experiments were approved by the Lower Saxony Office for Consumer Protection and Food Safety (LAVES; approval number 33.19-42502-04-20/3385), and performed according to the German animal welfare act and EU Directive 2010/63. Seven- to 8-week-old female NSG mice were injected with 5 × 10^5^ ffluc^+^ HB-82 cells into the tail vein. After 2 days, mice were injected with 5 × 10^5^ CORA_sh_mod-Ts or 5 × 10^5^ untransduced T cells into the tail vein. Respective mice injected with ffluc^+^ HB-82 cells but not treated with T cells served as controls. For *in vivo* imaging under anesthesia with isoflurane, mice were administered 150 mg/kg of D-luciferin (PerkinElmer, Waltham, MA, USA) in PBS by subcutaneous injection into the neck and, after 10 min, monitored using the IVIS SpectrumCT imaging system (Revvity, Waltham, MA, USA). Bioluminescence imaging was performed weekly, starting 1 week before ffluc^+^ HB-82 injection (w-1). Images in w0 were taken 15 to 20 min after injection of ffluc^+^ HB-82 cells to confirm similar bioluminescence signals of HB-82 cells in circulation between groups. Quantification of bioluminescence was performed by setting a defined rectangle area on each animal to obtain average radiance values using the Living Image 4.7 software (Revvity).

### Statistical analysis

Statistical analysis was performed by GraphPad Prism 9.5.1 using Mann-Whitney t tests or two-way ANOVA with Dunnet’s or Tukey’s multiple comparisons tests as indicated. ns: not significant, ∗*p* ≤ 0.05; ∗∗*p* ≤ 0.01; ∗∗∗*p* ≤ 0.001; ∗∗∗∗*p* ≤ 0.0001.

## Data and code availability

Data are available upon reasonable request. The datasets and protocols used and/or analyzed during the current study are available from the corresponding author (eiz-vesper.britta@mh-hannover.de) on reasonable request.

## Acknowledgments

We are grateful for the funding provided by 10.13039/501100003042Else Kröner-Fresenius-Stiftung (EKEA.174), nextGENERATION Medical Scientist Program funded by 10.13039/501100003042Else Kroner-Fresenius Foundation (2022_EKMK.13), Deutsche Krebshilfe/German Cancer Aid-Priority Program in Translational Oncology (111975) and 10.13039/501100007311Deutsche Kinderkrebsstiftung (DKS 2020.17). The author J.-P.G. was financially supported by Hannover Biomedical Research School. Moreover, we thank Elvira Schulde, Dörthe Rokitta, Sarina Lukis, Dr. Anja Battermann, Dr. Nadine Wenzel, and Henrike Voβ (all Institute of Transfusion Medicine and Transplant Engineering, MHH, Hannover, Germany) for their technical support. Moreover, we would like to thank Philipp Schleumann (imusyn GmbH & Co. KG, Hannover, Germany) for providing the eukaryotic HLA-A∗02 multimer. We also thank Prof. Dr. Axel Schambach, Prof. Dr. Christopher Baum, and Prof. Dr. Michael Morgan (all Institute of Experimental Hematology, MHH, Hannover) for providing the mCherry-encoding lentivirus, as well as Prof. Dr. Thomas Pietschmann and Vanessa Milke (both from TWINCORE, Center of Experimental and Clinical Infection Research, Hannover) for the opportunity to use the Incucyte SX5 Live-Cell Analysis System (Sartorius). Last, we thank Dr. Barbara Uchanska-Ziegler (Ziegler Biosolutions, Waldshut-Tiengen) and Prof. Dr. Martin Messerle (Institute of Virology, MHH, Hannover) for providing the TÜ165 and HFF cell lines, respectively.

## Author contributions

Conceptualization: A.C.D., C.F., R.B., and B.E.-V.; Data curation: A.C.D., A.B., M.V., and J.-P.G.; Formal analysis: A.C.D.; Funding acquisition: A.C.D., R.B., and B.E.-V.; Investigation: A.C.D., A.B., S.L., M.V., and J.-P.G.; Methodology: A.C.D., A.B., S.L., M.V., J.-P.G., F.I., and C.H.; Project administration: A.C.D., R.B., and B.E.-V.; Software: A.C.D., M.V., and J.-P.G.; Resources: A.C.D., R.B., and B.E.-V.; Supervision: M.H., C.F., R.B., and B.E.-V.; Validation: A.C.D., A.B., and B.E.-V.; Visualization: A.C.D.; Writing – original draft: A.C.D.; Writing – review & editing: A.C.D., A.B., M.V., J.-P.G., F.I., C.H., M.H., C.F., R.B., and B.E.-V.

## Declaration of interests

The authors declare no competing interests, except that authors A.C.D., C.F., R.B., and B.E.-V. are inventors of a patent describing the CORA-T approach (EP 3733697, WO 2020/221902: Artificial signaling molecule).

## Supplemental information

Document S1. Figures S1–S10 and Table S1

Document S2. Article plus supplemental information

## References

[1] Chong A.S., Rothstein D.M., Safa K., Riella L.V. (2019). Outstanding questions in transplantation: B cells, alloantibodies, and humoral rejection. Am. J. Transpl..

[2] Hart A., Singh D., Brown S.J., Wang J.H., Kasiske B.L. (2021). Incidence, risk factors, treatment, and consequences of antibody-mediated kidney transplant rejection: A systematic review. Clin. Transpl..

[3] Böhmig G.A., Eskandary F., Doberer K., Halloran P.F. (2019). The therapeutic challenge of late antibody-mediated kidney allograft rejection. Transpl. Int..

[4] Valenzuela N.M., Reed E.F. (2017). Antibody-mediated rejection across solid organ transplants: manifestations, mechanisms, and therapies. J. Clin. Invest..

[5] Schlößer H.A., Thelen M., Dieplinger G., von Bergwelt-Baildon A., Garcia-Marquez M., Reuter S., Shimabukuro-Vornhagen A., Wennhold K., Haustein N., Buchner D. (2017). Prospective Analyses of Circulating B Cell Subsets in ABO-Compatible and ABO-Incompatible Kidney Transplant Recipients. Am. J. Transpl..

[6] Melenhorst J.J., Chen G.M., Wang M., Porter D.L., Chen C., Collins M.A., Gao P., Bandyopadhyay S., Sun H., Zhao Z. (2022). Decade-long leukaemia remissions with persistence of CD4(+) CAR T cells. Nature.

[7] Ius F., Verboom M., Sommer W., Poyanmehr R., Knoefel A.K., Salman J., Kuehn C., Avsar M., Siemeni T., Erdfelder C. (2018). Preemptive treatment of early donor-specific antibodies with IgA- and IgM-enriched intravenous human immunoglobulins in lung transplantation. Am. J. Transpl..

[8] Altermann W.W., Seliger B., Sel S., Wendt D., Schlaf G. (2006). Comparison of the established standard complement-dependent cytotoxicity and flow cytometric crossmatch assays with a novel ELISA-based HLA crossmatch procedure. Histol. Histopathol..

[9] Jutz S., Leitner J., Schmetterer K., Doel-Perez I., Majdic O., Grabmeier-Pfistershammer K., Paster W., Huppa J.B., Steinberger P. (2016). Assessment of costimulation and coinhibition in a triple parameter T cell reporter line: Simultaneous measurement of NF-κB, NFAT and AP-1. J. Immunol. Methods.

[10] Purbhoo M.A., Boulter J.M., Price D.A., Vuidepot A.L., Hourigan C.S., Dunbar P.R., Olson K., Dawson S.J., Phillips R.E., Jakobsen B.K. (2001). The human CD8 coreceptor effects cytotoxic T cell activation and antigen sensitivity primarily by mediating complete phosphorylation of the T cell receptor zeta chain. J. Biol. Chem..

[11] Glienke W., Dragon A.C., Zimmermann K., Martyniszyn-Eiben A., Mertens M., Abken H., Rossig C., Altvater B., Aleksandrova K., Arseniev L. (2022). GMP-Compliant Manufacturing of TRUCKs: CAR T Cells targeting GD(2) and Releasing Inducible IL-18. Front. Immunol..

[12] Sommermeyer D., Hudecek M., Kosasih P.L., Gogishvili T., Maloney D.G., Turtle C.J., Riddell S.R. (2016). Chimeric antigen receptor-modified T cells derived from defined CD8+ and CD4+ subsets confer superior antitumor reactivity in vivo. Leukemia.

[13] Amini L., Wagner D.L., Rössler U., Zarrinrad G., Wagner L.F., Vollmer T., Wendering D.J., Kornak U., Volk H.D., Reinke P., Schmueck-Henneresse M. (2021). CRISPR-Cas9-Edited Tacrolimus-Resistant Antiviral T Cells for Advanced Adoptive Immunotherapy in Transplant Recipients. Mol. Ther..

[14] Hsiao C.Y., Ho M.C., Ho C.M., Wu Y.M., Lee P.H., Hu R.H. (2021). Long-Term Tacrolimus Blood Trough Level and Patient Survival in Adult Liver Transplantation. J. Pers. Med..

[15] Globerson Levin A., Rivière I., Eshhar Z., Sadelain M. (2021). CAR T cells: Building on the CD19 paradigm. Eur. J. Immunol..

[16] Walker A.J., Majzner R.G., Zhang L., Wanhainen K., Long A.H., Nguyen S.M., Lopomo P., Vigny M., Fry T.J., Orentas R.J., Mackall C.L. (2017). Tumor Antigen and Receptor Densities Regulate Efficacy of a Chimeric Antigen Receptor Targeting Anaplastic Lymphoma Kinase. Mol. Ther..

[17] Gao G.F., Tormo J., Gerth U.C., Wyer J.R., McMichael A.J., Stuart D.I., Bell J.I., Jones E.Y., Jakobsen B.K. (1997). Crystal structure of the complex between human CD8alpha(alpha) and HLA-A2. Nature.

[18] Zachary A.A., Kopchaliiska D., Montgomery R.A., Leffell M.S. (2007). HLA-specific B cells: I. A method for their detection, quantification, and isolation using HLA tetramers. Transplantation.

[19] Dragon A.C., Bonifacius A., Verboom M., Hudecek M., Figueiredo C., Blasczyk R., Eiz-Vesper B. (2023). Depletion of alloreactive B cells by chimeric alloantigen receptor T cells with drug resistance to prevent antibody-mediated rejection in solid organ transplantation. bioRxiv.

[20] Gille I., Hagedoorn R.S., van der Meer-Prins E.M.W., Heemskerk M.H.M., Heidt S. (2023). Chimeric HLA antibody receptor T cells to target HLA-specific B cells in solid organ transplantation. Hla.

[21] Betriu S., Rovira J., Arana C., García-Busquets A., Matilla-Martinez M., Ramirez-Bajo M.J., Bañon-Maneus E., Lazo-Rodriguez M., Bartoló-Ibars A., Claas F.H.J. (2023). Chimeric HLA antibody receptor T cells for targeted therapy of antibody-mediated rejection in transplantation. Hla.

[22] Mackensen A., Müller F., Mougiakakos D., Böltz S., Wilhelm A., Aigner M., Völkl S., Simon D., Kleyer A., Munoz L. (2022). Anti-CD19 CAR T cell therapy for refractory systemic lupus erythematosus. Nat. Med..

[23] Müller F., Taubmann J., Bucci L., Wilhelm A., Bergmann C., Völkl S., Aigner M., Rothe T., Minopoulou I., Tur C. (2024). CD19 CAR T-Cell Therapy in Autoimmune Disease - A Case Series with Follow-up. N. Engl. J. Med..

[24] Eskandary F., Regele H., Baumann L., Bond G., Kozakowski N., Wahrmann M., Hidalgo L.G., Haslacher H., Kaltenecker C.C., Aretin M.B. (2018). A Randomized Trial of Bortezomib in Late Antibody-Mediated Kidney Transplant Rejection. J. Am. Soc. Nephrol..

[25] Kwun J., Burghuber C., Manook M., Iwakoshi N., Gibby A., Hong J.J., Knechtle S. (2017). Humoral Compensation after Bortezomib Treatment of Allosensitized Recipients. J. Am. Soc. Nephrol..

[26] Ellebrecht C.T., Bhoj V.G., Nace A., Choi E.J., Mao X., Cho M.J., Di Zenzo G., Lanzavecchia A., Seykora J.T., Cotsarelis G. (2016). Reengineering chimeric antigen receptor T cells for targeted therapy of autoimmune disease. Science.

[27] Lee J., Lundgren D.K., Mao X., Manfredo-Vieira S., Nunez-Cruz S., Williams E.F., Assenmacher C.A., Radaelli E., Oh S., Wang B. (2020). Antigen-specific B cell depletion for precision therapy of mucosal pemphigus vulgaris. J. Clin. Invest..

[28] Parvathaneni K., Scott D.W. (2018). Engineered FVIII-expressing cytotoxic T cells target and kill FVIII-specific B cells in vitro and in vivo. Blood Adv..

[29] Oh S., Mao X., Manfredo-Vieira S., Lee J., Patel D., Choi E.J., Alvarado A., Cottman-Thomas E., Maseda D., Tsao P.Y. (2023). Precision targeting of autoantigen-specific B cells in muscle-specific tyrosine kinase myasthenia gravis with chimeric autoantibody receptor T cells. Nat. Biotechnol..

[30] Reincke S.M., von Wardenburg N., Homeyer M.A., Kornau H.C., Spagni G., Li L.Y., Kreye J., Sánchez-Sendín E., Blumenau S., Stappert D. (2023). Chimeric autoantibody receptor T cells deplete NMDA receptor-specific B cells. Cell.

[31] Dragon A.C., Zimmermann K., Nerreter T., Sandfort D., Lahrberg J., Klöß S., Kloth C., Mangare C., Bonifacius A., Tischer-Zimmermann S. (2020). CAR-T cells and TRUCKs that recognize an EBNA-3C-derived epitope presented on HLA-B∗35 control Epstein-Barr virus-associated lymphoproliferation. J. Immunother. Cancer.

[32] Brinkman E.K., Chen T., Amendola M., van Steensel B. (2014). Easy quantitative assessment of genome editing by sequence trace decomposition. Nucleic Acids Res..

