# Supplementary material for: Depletion of alloreactive B cells by drug-resistant chimeric alloantigen receptor T cells to prevent transplant rejection
Source: Mol Ther. 2025 Jan 11;33(3):1031–47. doi: 10.1016/j.ymthe.2025.01.009 (PMC11897811; doi:10.1016/j.ymthe.2025.01.009)
Supplement: Document S1. Figures S1–S10 and Table S1 [file mmc1.pdf]

YMTHE, Volume *33*

Supplemental Information

Depletion of alloreactive B cells by drug-resistant chimeric alloantigen receptor T cells to prevent transplant rejection

Anna Christina Dragon, Agnes Bonifacius, Stefan Lienenklaus, Murielle Verboom, Jan-Phillipp Gerhards, Fabio Ius, Christian Hinze, Michael Hudecek, Constanca Figueiredo, Rainer Blasczyk, and Britta Eiz-Vesper

SUPPLEMENTAL FIGURES

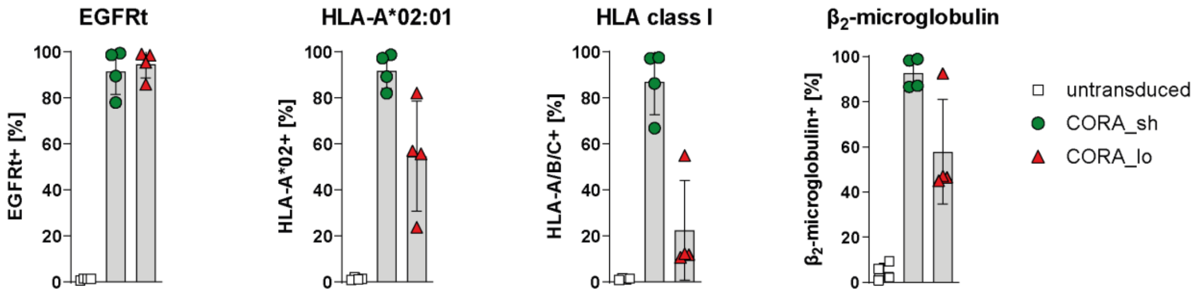

Fig. S1: CORA receptors harbor a truncated HLA-A*02 as recognition domain. CORA_sh and CORA_lo receptors were generated and expressed in SPI-801 cells by lentiviral transduction. Expression of EGFRt as marker for transduced cells, HLA-A*02, HLA class I and β_2-microglobulin was assessed by flow cytometry. Untransduced SPI-801 cells served as control. Data are shown as scattered dot plot with mean±SD, whereby each symbol represents an independent experiment (n=4).

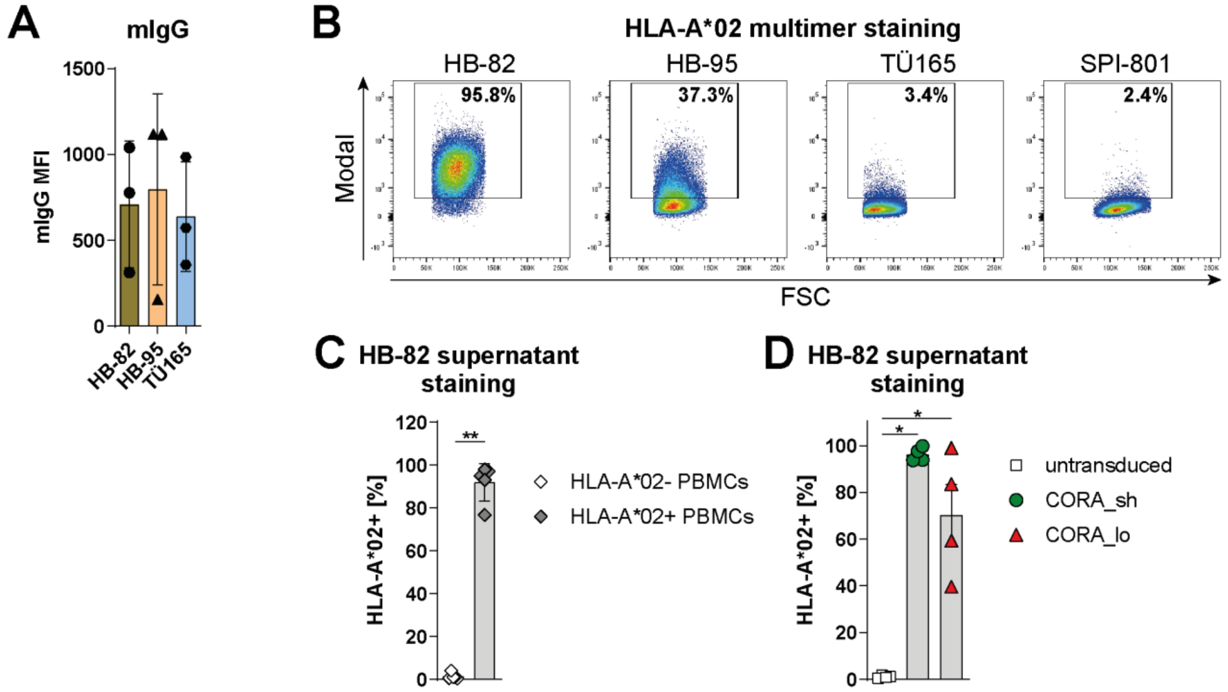

Fig. S2: Hybridoma cells serve as model for anti-HLA-A*02 B cells releasing anti-HLA*A02 antibodies. HB-82 (anti-HLA-A*02), HB-92 (anti-HLA-A/B/C) and TÜ165 (anti-HLA-B*35 loaded with LPPHDITPY) cells were used as model for anti-HLA-antibody-releasing B cells. **(A)** Surface expression of murine BCRs was determined by flow cytometry and usage of anti-mouse immunoglobulin G (mIgG) antibody. **(B)** Recognition of HLA-A*02 by hybridoma cells was assessed by staining with eukaryotic HLA-A*02/NLV multimer. **(C, D)** Cell culture supernatant of HB-82 cells containing anti-HLA-A*02 antibody was used to stain **(C)** HLA-A*02-negative or -positive PBMCs from healthy donors, as well as **(D)** SPI-801 cells transduced with CORA_sh or CORA_lo receptors. **(A, C-D)** Data are shown as scattered dot plot with mean±SD, whereby each symbol represents an independent experiment (n=3-5). Statistical analysis was performed by using Mann-Whitney test. *p≤0.05, **p≤0.01.

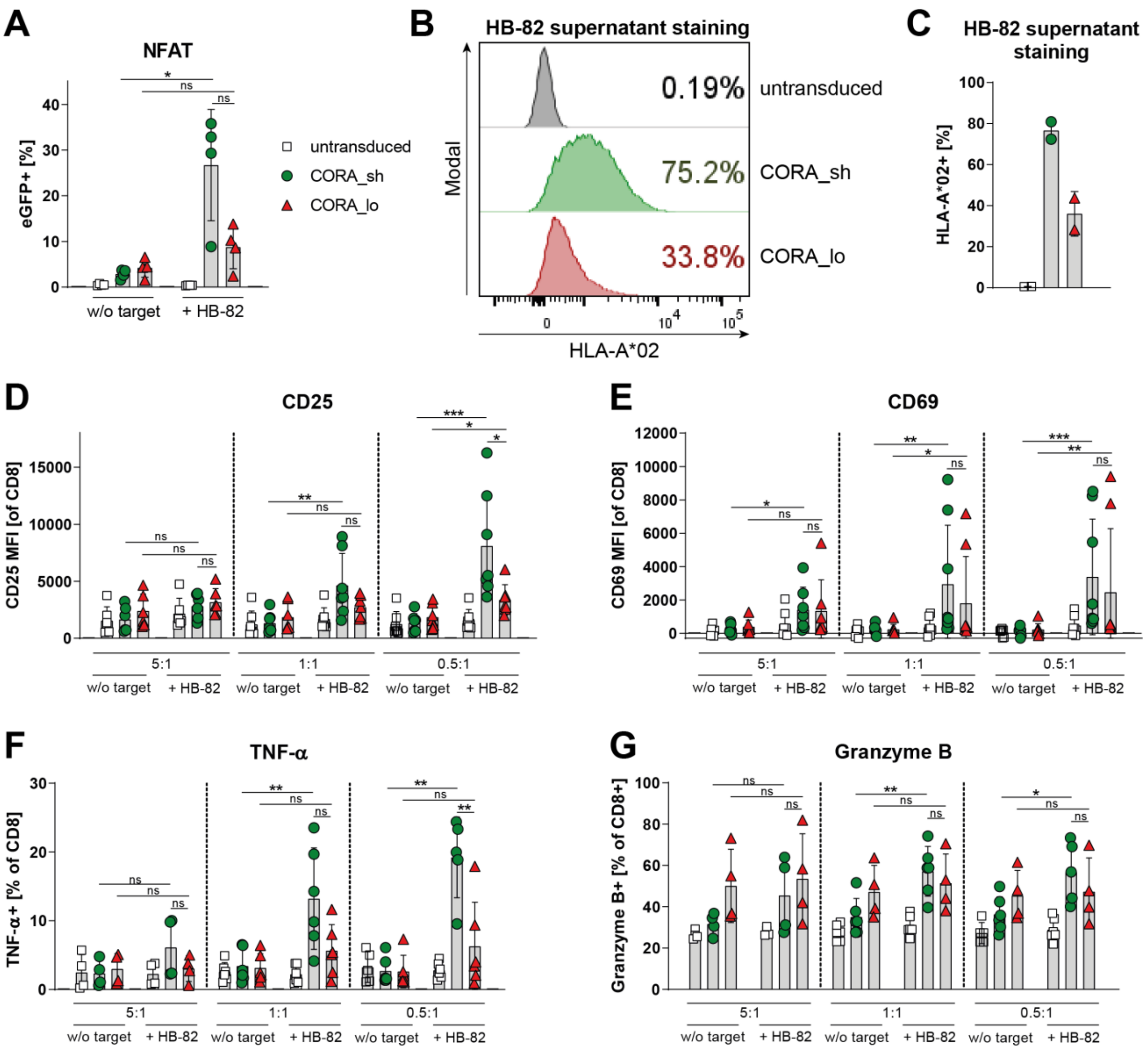

Fig. S3: CORA receptors are detectable on the cell surface after transduction into primary CD8+ T cells and mediate effective and target-specific T-cell signaling, activation and cytokine expression. CORA receptors with either a short (CORA_sh) or long (CORA_lo) spacer domain were transduced into (**A**) Jurkat-based reporter cells or (**B-G**) primary CD8+ T cells. Respective untransduced cells served as controls. (**A**) After cultivation of transduced reporter cells without (w/o) target cells or with HB-82 cells (anti-HLA-A*02) in an E:T ratio of 1:1 for 24 h, transcription factor activity was determined by evaluation of NFAT-induced enhanced green fluorescent protein (eGFP) reporter expression by flow cytometry (n=4). (**B, C**) After manufacturing of CORA-Ts from primary CD8+ T cells, HLA-A*02 expression on the cell surface was assessed by flow cytometry and staining with cell culture supernatant of HB-82 cells containing anti-HLA-A*02 antibody. Data are shown as **(B)** representative histograms or **(C)** scattered dot plot with mean±SD, whereby each symbol represents an independent donor (n=2).

(D-G) After co-cultivation of transduced $CD8^+$ T cells with HB-82 cells in the indicated E:T ratios for 48 h, expression of **(D, E)** activation markers (n=6-8) and **(F, G)** intracellular cytokines (n=4-6) was assessed by flow cytometry. **(A, C-G)** Data are shown as scattered dot plot with mean±SD, whereby each symbol represents an independent donor. Statistical analysis was performed by using Mann-Whitney test. ns: not significant, *p≤0.05, **p≤0.01, ***p≤0.001.

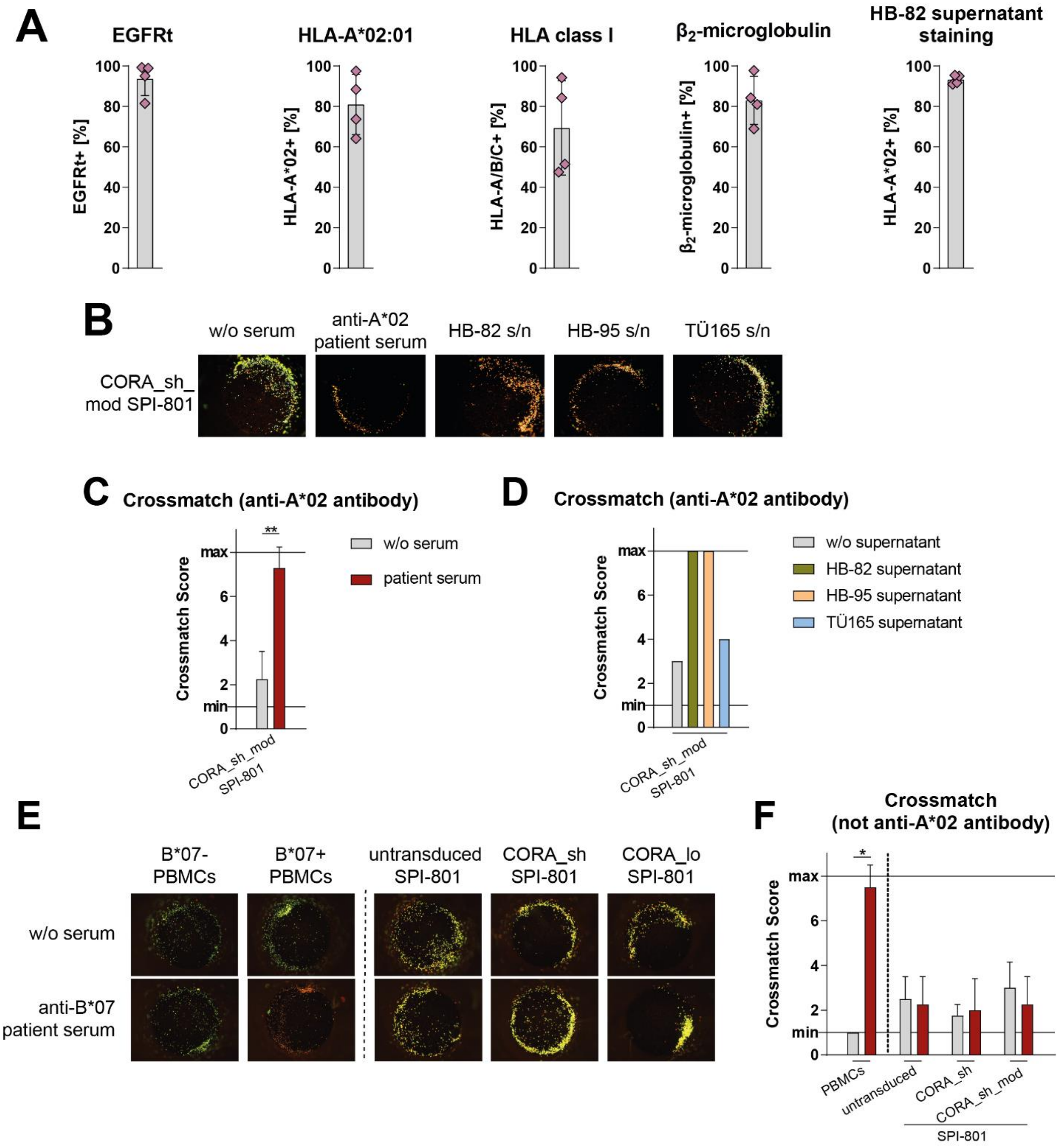

Fig. S4: Modification of the HLA-A*02 component of CORA receptors to abrogate CD8 binding does not interfere with HLA complex association nor recognition by antibodies. CORA receptors comprising a modified truncated HLA-A*02 molecule (D227K, T228A) and a short spacer domain (CORA_sh_mod) were transduced into SPI-801 cells. **(A)** Expression of EGFRt, HLA-A*02, HLA class I and β_2-microglobulin was assessed by flow cytometry. Moreover, cell culture supernatant of HB-82 cells containing anti-HLA-A*02 antibody was used to stain CORA_sh_mod$^+$ SPI-801 cells (right graph). Data are shown as scattered dot plot with mean±SD, whereby each symbol represents an independent experiment (n=4). **(B-D)** Binding of

anti-HLA-A*02 antibodies present in **(B, C)** the serum of kidney transplant recipients or **(B, D)** the supernatant (s/n) of hybridoma cells to CORA_sh_mod$^+$ SPI-801 cells was assessed by their ability to mediate complement-dependent cytotoxicity (CDC) in crossmatch assays. (**E, F**) As control, crossmatch assays were performed, in which CORA_sh$^+$ and CORA_sh_mod$^+$ SPI-801 were incubated with the serum of HLA-sensitized patients that contained (**E**), among, others, anti-HLA-B*07 antibodies or (**F**) various other HLA antibodies except anti-HLA-A*02 antibodies. PBMCs positive for at least one of these HLA molecules served as control. **(B, E)** Representative pictures and **(C, D, F)** crossmatch scores indicate CDC based on evaluation of viable cells (green) versus dead cells (red) after complement addition. Respective cells incubated without (w/o) supernatant served as viable controls. **(C, F)** Data are shown as mean+SD (n=4-10). Statistical analysis was performed by using Mann-Whitney test. *$p \leq 0.05$.

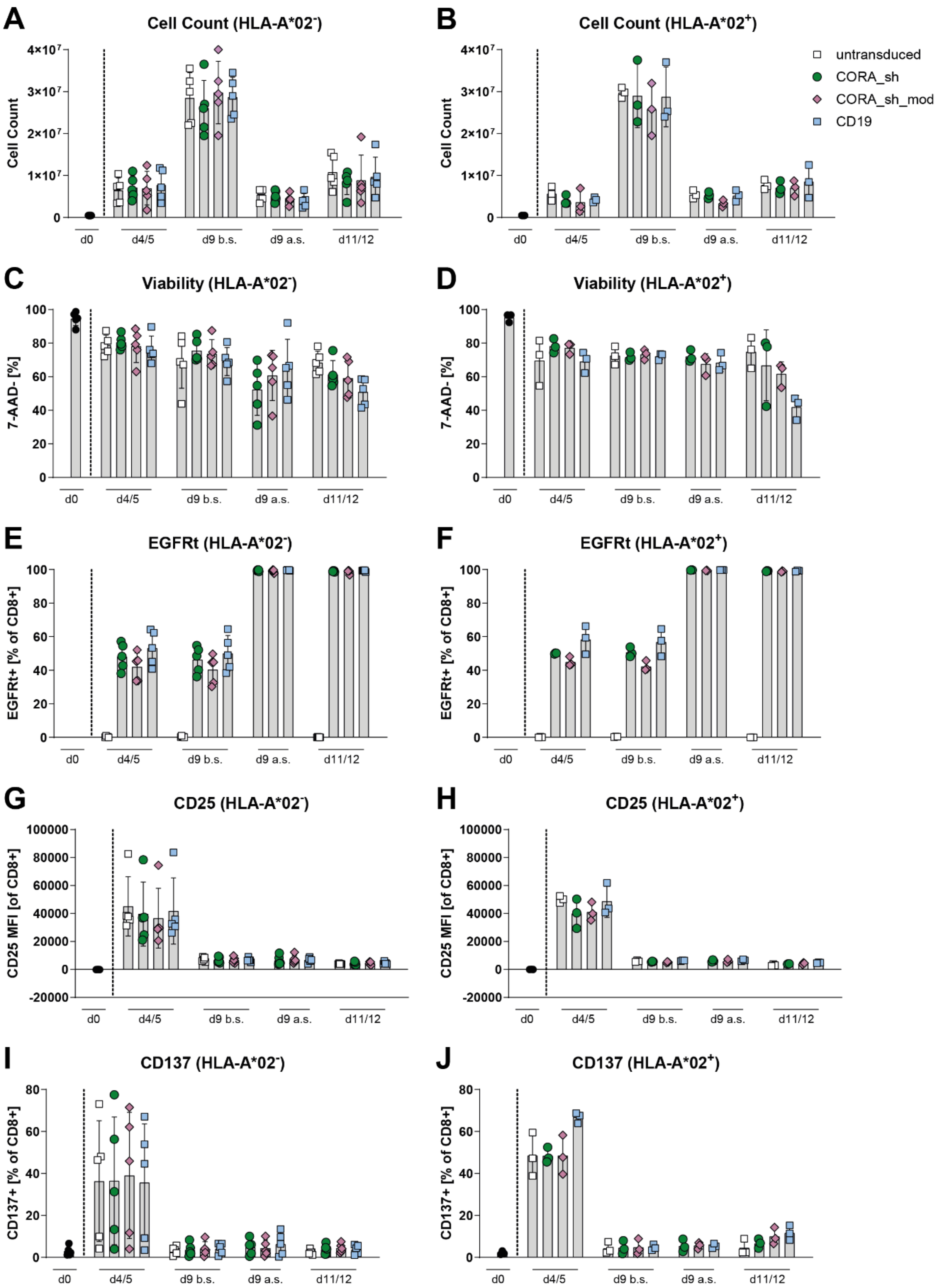

Fig. S5: Transduction of CD8⁺ T cells with CORA receptors does not cause fratricide. CORA_sh receptors comprising either a truncated wildtype or a modified (CORA_sh_mod) HLA-A*02 molecule were transduced into primary CD8⁺ T cells isolated from healthy (**A, C, E, G, I**)

HLA-A*02-negative (n=5) or (**B, D, F, H, J**) HLA-A*02-positive (n=3) donors. As control, T cells from the same donors were transduced with a CD19-targeting CAR using the same protocol. Transduced cells were enriched using EGFRt on day (d) 9. Moreover, untransduced T cells expanded analogously without addition of lentivirus and enrichment served as controls. **(A, B)** During expansion, as well as before sorting (b.s.) and after sorting (a.s.) on d9, viable cells were counted with trypan blue exclusion. (**B-J**) At the same time points, T cells were evaluated using flow cytometry to determine (**C, D**) viability, (**E, F**) frequency of transduced cells, and (**G-J**) activation states. Data are shown as scattered dot plot with mean±SD, whereby each symbol represents one donor.

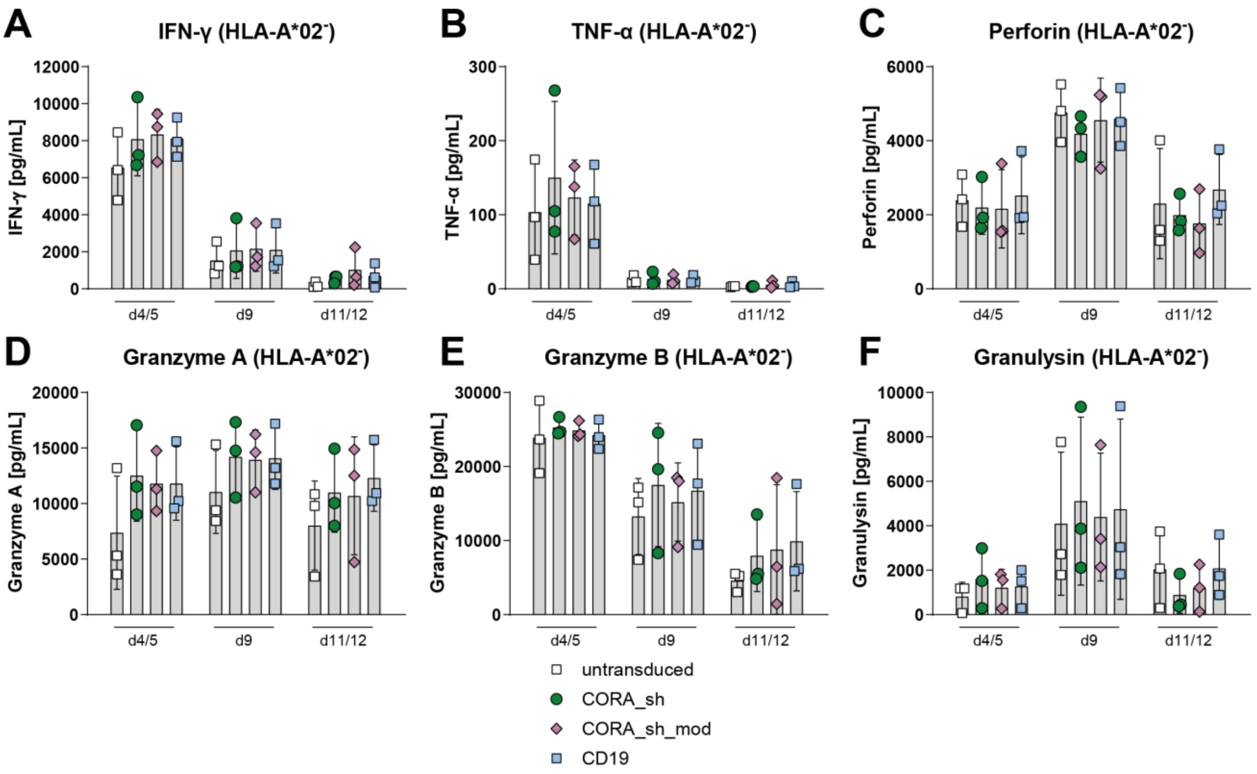

Fig. S6: Transduction of CD8+ T cells with CORA receptors does not cause cytokine and cytotoxic mediator release. CORA_sh receptors comprising either a truncated wildtype or a modified (CORA_sh_mod) HLA-A*02 molecule were transduced into primary CD8+ T cells isolated from healthy HLA-A*02-negative donors. As control, T cells from the same donors were transduced with a CD19-targeting CAR using the same protocol. Transduced cells were enriched using EGFRt on day (d) 9. Moreover, untransduced T cells expanded analogously without addition of lentivirus and enrichment served as controls. At the indicated time points of expansion, supernatants of T-cell cultures were evaluated for the presence of cytokines and cytotoxic mediators using LEGENDplex. Data are shown as scattered dot plot with mean±SD, whereby each symbol represents one donor.

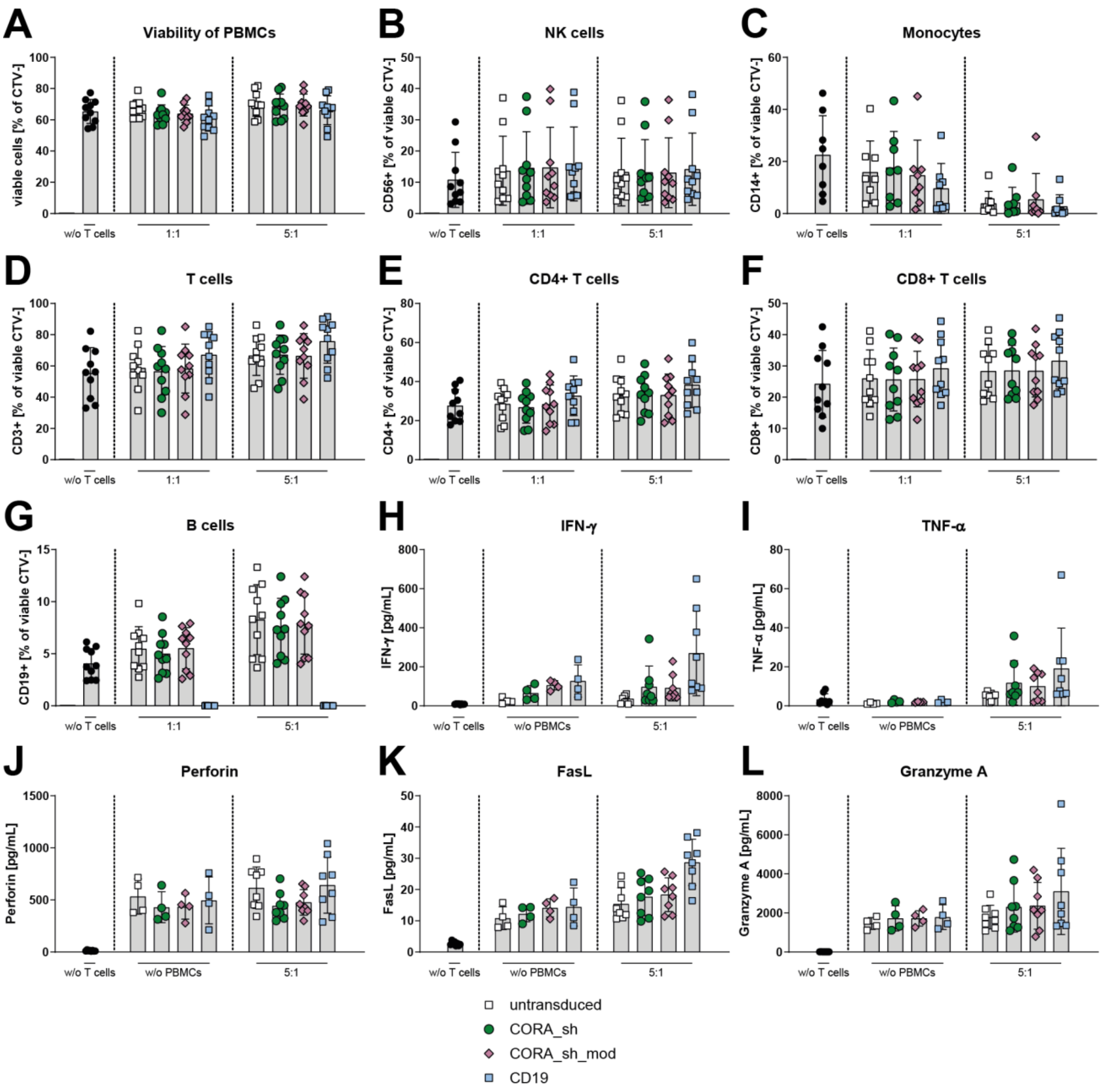

Fig. S7: CORA_sh- and CORA_sh_mod-Ts do not mediate unintended elimination of other immune cells. CORA_sh- and CORA_sh_mod-Ts were generated from $CD8^+$ T cells of healthy, not HLA-sensitized donors, enriched using EGFRt on day 9 and frozen on day 11-12. CD19-CAR-Ts or untransduced T cells expanded analogously served as controls. They were thawed, rested for one day in T-cell medium supplemented with 12.5 ng/mL IL-7 and IL-15 and then co-cultured with autologous PBMCs in the indicated E:T ratios for 48 h. Data are shown as scattered dot plot with mean±SD, whereby each symbol represents one replicate (n=8-10 from 4-5 independent donors). Prior to the co-culture, T cells were labeled with CTV to determine (**A**) the frequency of living PBMCs as CTV^- $7\text{-}AAD^-$ and (**B-G**) frequencies of the indicated immune cells among CTV^- PBMCs after co-culture using flow cytometry. As control, viability and immune cell frequencies were determined in cultures of PBMCs without (w/o) T cells. (**H-L**) Release of soluble mediators

into the supernatant was assessed by LEGENDplex, whereby PBMCs cultured w/o T cells and T cells cultured w/o PBMCs served as controls.

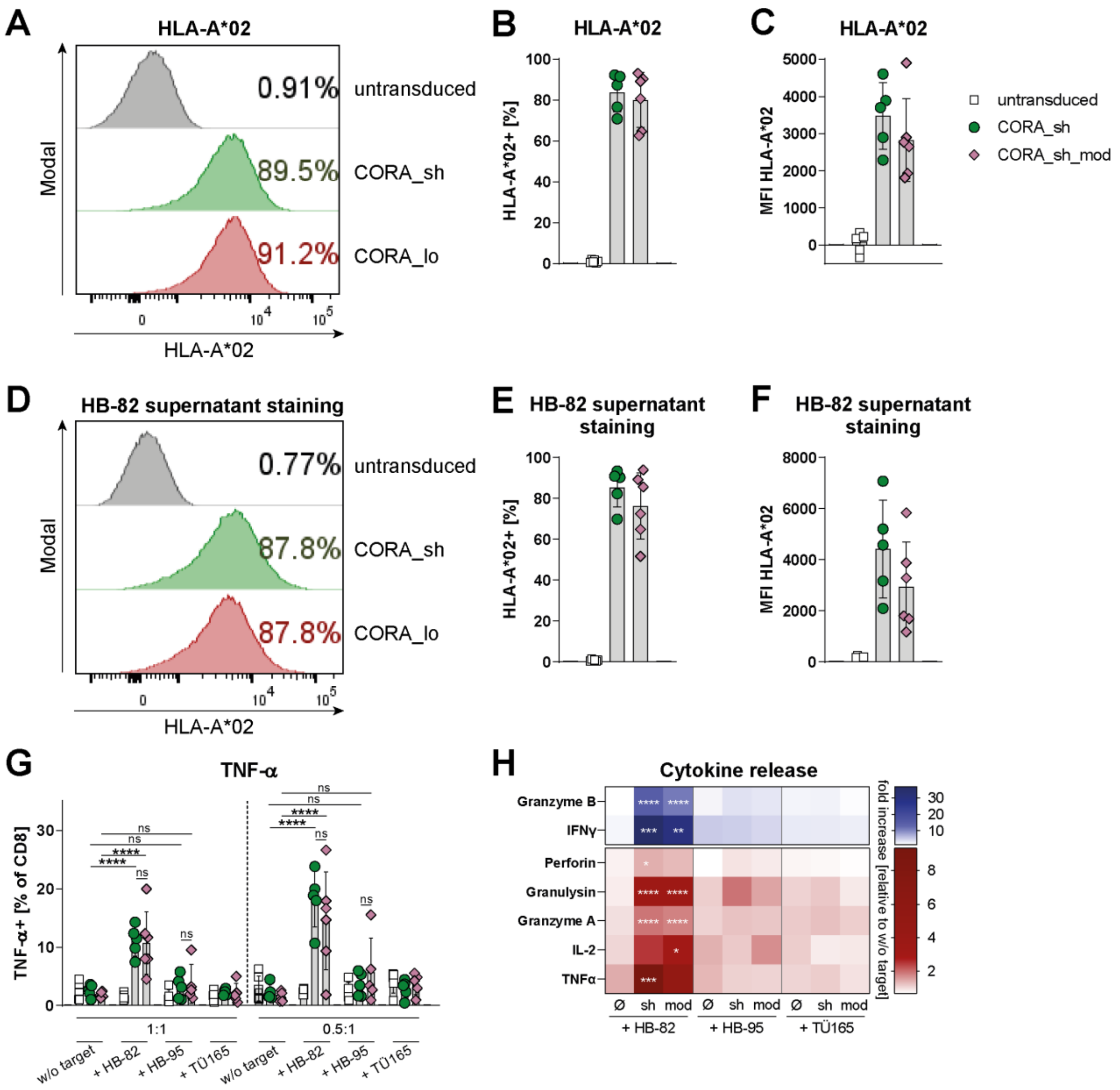

Fig. S8: CORA-Ts with a truncated HLA-A*02 molecule as recognition domain exhibit effective and target-specific cytokine expression, which is unaltered by modification for abrogation of T-cell-sensitization. CORA_sh receptors comprising either a truncated wildtype or a modified (CORA_sh_mod) HLA-A*02 molecule were transduced into primary CD8$^+$ T cells isolated from healthy donors. Untransduced T cells served as controls. **(A-F)** The HLA-A*02 component of the CORA receptor was detected by flow cytometry and staining with **(A-C)** an anti-HLA-A*02 antibody or **(D-F)** cell culture supernatant of HB-82 cells. Expression is shown as **(A, D)** representative histograms, **(B, E)** frequency (n=5-6) or **(C, F)** MFI (n=5-6) of CD8$^+$ T cells. **(G, H)** Generated CORA-Ts were cultured without (w/o) target cells or with the indicated target cells in **(G)** the indicated effector-to-target (E:T) ratio or **(H)** an E:T ratio of 5:1 for 48 h. **(G)** Expression of intracellular cytokines in CD8$^+$ T cells was evaluated by flow cytometry. Data are

shown as scattered dot plot with mean±SD, whereby each symbol represents an independent donor (n=5-6). Statistical analysis was performed by using Two-Way ANOVA with Tukey's multiple comparisons test. ns: not significant, *p≤0.05, **p≤0.01, ****p≤0.0001. **(H)** Release of cytokines and cytotoxic mediators by untransduced (ø) and transduced $CD8^+$ T cells into the supernatant was assessed by LEGENDplex. Fold increase to respective T cells cultures w/o target is shown as mean (n=7-8).

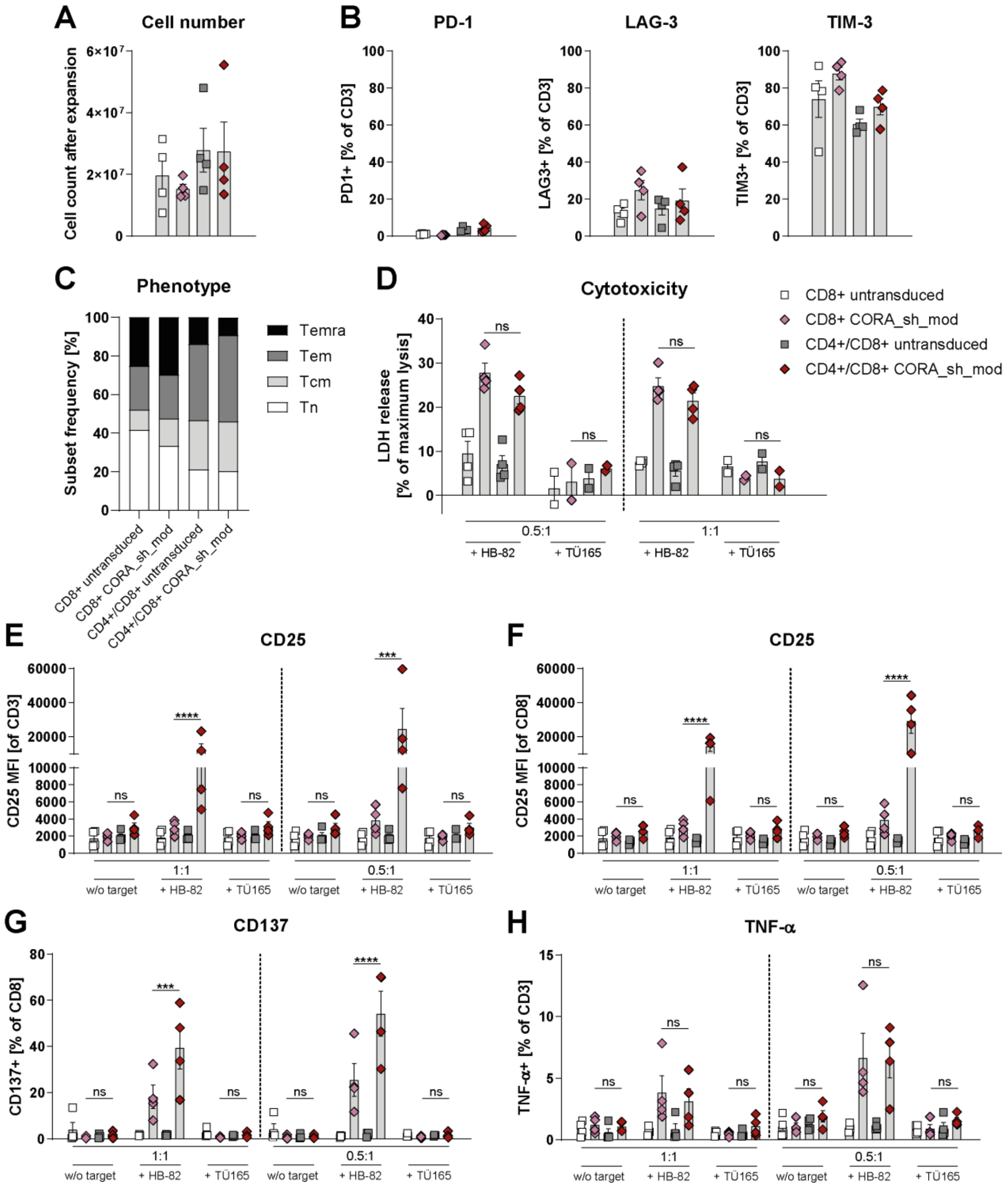

Fig. S9: CORA-Ts generated using a protocol similar to clinical manufacturing exhibit similar *in vitro* properties but mediate higher T-cell activation following target recognition. CORA_sh_mod-Ts were generated by using a small-scale protocol similar to the manufacturing of CAR-Ts at the CliniMACS Prodigy® using $CD4^+$ and $CD8^+$ T cells as starting material ($CD4^+/CD8^+$ CORA_sh_mod) and compared to CORA_sh_mod-Ts manufactured using the

previous protocol based on transduction of $CD8^+$ T cells ($CD8^+$ CORA_sh_mod). Respective untransduced T cells served as controls. After manufacturing, **(A)** cell numbers were counted and **(B)** markers for exhaustion and **(C)** memory phenotypes evaluated using flow cytometry (n=4). Phenotypes were differentiated between naïve T cells (Tn; $CD45RA^+CD62L^+$), central memory T cells (Tcm; $CD45RA^-CD62L^+$), effector memory T cells (Tem; $CD45RA^-CD62L^-$), and effector memory T cells re-expressing CD45RA (Temra; $CD45RA^+CD62L^-$) **(D-H)** Generated CORA-Ts were cultured without (w/o) target cells or with the indicated target cells in the indicated effector-to-target (E:T) ratios for 48 h. **(D)** Co-culture supernatants were evaluated for levels of lactate dehydrogenase (LDH) as indicator for T-cell mediated cytotoxicity (n=2-4). Expression of **(E-G)** activation markers and **(H)** intracellular TNF-α was evaluated by flow cytometry and gating on **(E,H)** $CD3^+$ or **(F-G)** $CD8^+$ T cells (n=4). Data are shown as **(A,B,D-H)** scattered dot plot with mean+SD, whereby each symbol represents an independent donor or **(C)** mean. **(D-H)** Statistical analysis was performed by using Two-Way ANOVA with Tukey's multiple comparisons test. ns: not significant, ***$p \leq 0.001$, ****$p \leq 0.0001$.

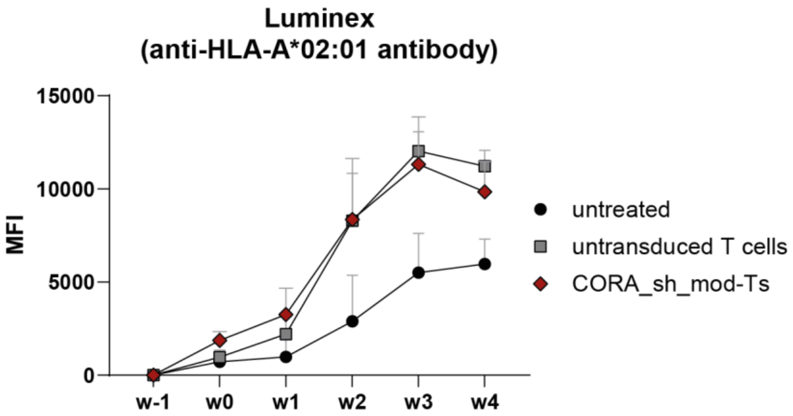

Fig. S10: Anti-HLA antibody are detectable in the blood of mice. Female NSG mice were injected $5x10^5$ ffluc$^+$ HB-82 cells followed by injection of $5x10^5$ CORA_sh_mod-Ts or untransduced T cells two days later. Respective mice injected with ffluc$^+$ HB-82 cells but not treated with T cells (untreated) served as controls. Serum or plasma samples of mice were taken weekly, 1:4 diluted and evaluated for presence of anti-HLA-A*02:01 antibodies by Luminex. Data are shown as mean+SEM (n=5).

SUPPLEMENTAL METHODS

Construction of CORA receptors

Sequences of HLA-A*02:01:01:01 exons 1-4 (GenBank no. HG794376.1) were cloned into two previously described TÜ165-CAR-epHIV7 vectors by using NheI and RsrII restriction sites to, respectively, replace the signaling peptide and TÜ165 scFv.[1] Briefly, the resulting receptors comprised the extracellular chains α_1-α_3 of HLA-A*02:01 fused to either a short (CORA_sh) "hinge-only" (12 aa) or a long (CORA_lo) "hinge-CH2-CH3" (229 aa) spacer domain region derived from IgG4-Fc followed by a transmembrane domain (TMD) of CD28 and the intracellular signaling domains of 4-1BB and CD3ζ (Fig. 1A). EGFRt[2] was encoded in the same vector by using a self-cleaving T2A element and served as marker for detection and enrichment of transduced cells. A previously-described CD19-targeting CAR[3] with the same spacer, TMD, signaling and EGFRt domains served as control in some experiments.

Generation of lentivirus as vector for transfer of CORA receptors

CORA_sh, CORA_lo, CORA_sh_mod, CORA_lo_mod, and CD19-CAR lentiviral particles were produced similar as described before.[1] Briefly, 293T cells (ACC 635; DSMZ, Braunschweig, Germany) were transfected with respective CORA-epHIV7 vectors and third-generation packaging vectors in presence of 25 µM chloroquine. Supernatants containing lentivirus were harvested after 32 h and 48 h and concentrated via ultracentrifugation. Titers were determined by transduction of Jurkat cells (ACC 282; DSMZ) in presence of 5 µg/mL Polybrene Infection/Transfection Reagent (Merck, Darmstadt, Germany). After 48 h, transduction efficiencies assessed by staining of co-expressed EGFRt in flow cytometry were used to calculate virus titers.

Cell lines and co-cultures with CORA receptor-transduced SPI-801 cells

SPI-801 cells (ACC 86; DSMZ) were transduced with respective CORA receptors in a multiplicities of infections (MOI) of 1 and 5 µg/mL Polybrene Infection/Transfection Reagent (Merck). Cells harboring the receptor were enriched by using biotinylated anti-EGFR antibody and anti-biotin microbeads (Miltenyi Biotec, Bergisch Gladbach, Germany).

Abrogation of CD8 binding to the CORA_sh_mod receptor was evaluated by co-cultivation of irradiated CORA-receptor-transduced SPI-801 cells with $CD8^+$ T cells isolated from PBMCs of healthy HLA-A*02-positive individuals in an E:T ratio of 1:1 for 7 days. Peptide-loading of CORA receptor-transduced SPI-801 cells was performed in some of these experiments by adding the CMV-derived NLVPMVATV peptide (ProImmune, Oxford, UK) to respective cells in serum-free medium overnight before the co-culture. For evaluation of cytotoxicity towards $pp65_{NLV}$-loaded CORA receptor-transduced SPI-801 cells, HLA-A*02/$pp65_{NLV}$-specific T cells were enriched from $CD8^+$ T cells isolated from healthy HLA-A*02-positive individuals by staining with

APC-conjugated Dextramer HLA-A*02:01 (NLVPMVATV) (Immudex, Copenhagen, Denmark) and enrichment using anti-APC MicroBeads (Miltenyi).

The hybridoma cell lines HB-82 (anti-HLA-A*02; clone BB7.2; ATCC), HB-95 (anti-HLA class I; clone W6/32; ATCC) and TÜ165 (against HLA-B*35:01 loaded with LPPHDITPY; kindly provided by Dr. Barbara Uchanska-Ziegler (Ziegler Biosolutions, Waldshut-Tiengen, Germany [4])) were used as surrogates for B cells expressing anti-HLA BCRs and releasing the respective antibody. Cell culture supernatants containing respective anti-HLA antibodies were harvested from all hybridoma cells. W6/32 SPI-801 cells were generated by transduction of a CAR construct based on the scFv of the anti-HLA class I antibody W6/32 into SPI-801 cells by lentiviral transduction.

Ffluc^{+} HB-82 cells were generated by transduction with a ffluc-encoding lentivirus. mCherry^{+} HB-82 cells were generated by transduction with an mCherry-encoding lentivirus that was kindly provided by Prof. Dr. Michael Morgan (MHH, Hannover).

Human Foreskin Fibroblast (HFF) cells (PromoCell, Heidelberg, Germany) were used as scaffold for one live-cell imaging experiment and obtained as kind gift from Prof. Dr. Martin Messerle (MHH, Hannover).

Flow cytometry

Antibodies used for flow cytometry are listed in table S1. The anti-EGFRt antibody was purchased (Erbitux; ImClone Systems, New York, NY, USA) and coupled to biotin (Thermo Fisher Scientific, Waltham, MA, USA) for further use. Staining of HLA was performed by addition of human Fc block (BD, Franklin Lakes, NJ, US) for 10 min, followed by addition of respective anti-HLA antibodies. Staining of HLA-A*02:01 or CORA receptors with cell culture supernatant of HB-82 cells containing anti-HLA-A*02:01 antibody (mIgG2b) was performed using an anti-mouse IgG secondary antibody (Jackson Immunoresearch, West Grove, PA, USA) for detection. Hybridoma cell staining was performed using a eukaryotic HLA-A*02 multimer loaded with the CMV-derived peptide NLVPMVATV (kindly provided by Philip Schleumann, Imusyn, Hannover, Germany). To determine frequencies of T cells specific for the HLA-A*02/pp65$_{NLV}$ complex, APC- or PE-conjugated Dextramer HLA-A*02:01 (NLVPMVATV) (Immudex, Copenhagen, Denmark) was used. T-cell proliferation was assessed by labeling CORA-Ts with CTV before co-culture with target cells and gating on CTV^{low} cells. Samples were analyzed on a BD FACSCanto Flow Cytometer (BD). Data were analyzed using FlowJo v10.

Table S1. Antibodies used for flow cytometry. Phycoerythrin (PE), Peridinin-chlorophyll-protein (PerCP), fluorescein isothiocyanate (FITC), Alexa Fluor® (AF), allophycocyanin (APC), Brilliant Violet™ (BV).

**Specificity**	**Antibody Clone**	**Fluorophore**	**Supplier**
EGFRt	-	(biotin)	ImClone Systems
Streptavidin	-	PE, APC	Thermo Fisher Scientific, BioLegend
CD3	SK7, UCHT1, HIT3a	AF700, PerCP, FITC	BioLegend
CD4		BV510	BioLegend
CD8	SK1	BV510, AF700, APC	BioLegend
CD14	HCD14	PE-Cy7	BioLegend
CD19	HIB19	FITC	BioLegend
CD25	BC96	APC-Cy7, PE-Cy7, BV421	BioLegend
CD56	HCD56	PE	BioLegend
CD69	FN50	BV605	BioLegend
CD137	4B4-1	PE-Cy7, APC	BioLegend
CD45RA	HI100	FITC	BioLegend
CD62L	DREG-56	PE-Cy7	BioLegend
Granzyme B	GB11	Pacific Blue	BioLegend
HLA class I	W6/32	FITC	Bio-Rad, Hercules, CA, USA
HLA-A*02	BB7.2	PerCP/Cy5.5	Biolegend
LAG-3	11C3C65	BV421	BioLegend
mouse IgG (H+L)	polyclonal $F(ab')_2$ fragment	PE	Jackson Immunoresearch
PD-1	EH12.2H7	BV605	BioLegend
TIM-3	F38-2E2	APC	BioLegend
TNF-a	MAb11	APC	BioLegend
β_2-microglobulin	2M2	PE	Biolegend

REFERENCES

1. Dragon, A.C., Zimmermann, K., Nerreter, T., Sandfort, D., Lahrberg, J., Kloss, S., Kloth, C., Mangare, C., Bonifacius, A., Tischer-Zimmermann, S., Blasczyk, R., et al. (2020). CAR-T cells and TRUCKs that recognize an EBNA-3C-derived epitope presented on HLA-B*35 control Epstein-Barr virus-associated lymphoproliferation. J Immunother Cancer 8. 10.1136/jitc-2020-000736.

2. Hudecek, M., Lupo-Stanghellini, M.T., Kosasih, P.L., Sommermeyer, D., Jensen, M.C., Rader, C., and Riddell, S.R. (2013). Receptor affinity and extracellular domain modifications affect tumor recognition by ROR1-specific chimeric antigen receptor T cells. Clin Cancer Res 19, 3153-3164. 10.1158/1078-0432.Ccr-13-0330.

3. Dragon, A.C., Beermann, L.M., Umland, M., Bonifacius, A., Malinconico, C., Ruhl, L., Kehler, P., Gellert, J., Weiß, L., Mayer-Hain, S., Zimmermann, K., et al. (2023). CAR-Ts redirected against the Thomsen-Friedenreich antigen CD176 mediate specific elimination of malignant cells from leukemia and solid tumors. Front Immunol 14, 1219165. 10.3389/fimmu.2023.1219165.

4. Uchańska-Ziegler, B., Nössner, E., Schenk, A., Ziegler, A., and Schendel, D.J. (1993). Soluble T cell receptor-like properties of an HLA-B35-specific monoclonal antibody (TU165). Eur J Immunol 23, 734-738. 10.1002/eji.1830230325.
